# *In silico* design and binding mechanism of UBR1 E3 ligase recruiters

**DOI:** 10.1039/d5ra04908c

**Published:** 2026-05-19

**Authors:** Miguel A. Maria-Solano, Raudah Lazim, Sun Choi

**Affiliations:** a Global AI Drug Discovery Center, College of Pharmacy and Graduate School of Pharmaceutical Science, Ewha Womans University 03760 Seoul Republic of Korea miguelangel.maria@uab.cat sunchoi@ewha.ac.kr

## Abstract

Proteolysis Targeting Chimeric Molecules (PROTACs) represent a promising avenue in drug discovery, as they can induce the targeted degradation of disease-relevant proteins within the cellular machinery. These compounds comprise a ligand tailored to bind the specific targeted protein connected to a recruiter molecule that engages with the E3 ligase. Despite their promise as therapeutic agents, the clinical advancement of these compounds has encountered substantial challenges, primarily due to the limited availability of suitable E3 ligases. Additionally, cell permeability and proteolytic stability, due to their peptide nature, often hinder their application. In this study, we developed a computational framework to model recruiters for the E3 ligase UBR1. This widely expressed protein has recently been demonstrated to be efficient in driving the degradation of oncogenic proteins. Our computational approach leverages a fragment-based peptidomimetics strategy, integrating pharmacophore filtering, docking, and fragment-linking optimization. Finally, we subjected the wild-type peptide and the most promising combined fragments to advanced binding free energy calculations, unveiling insights into their dynamic water-mediated binding mechanisms and their potential as robust E3 ligase UBR1 recruiters. This computational workflow is applicable to model other related PROTACs.

## Abstract 2

Virtual screening workflow for designing UBR1 E3 ligase recruiters.

## Graphical abstract

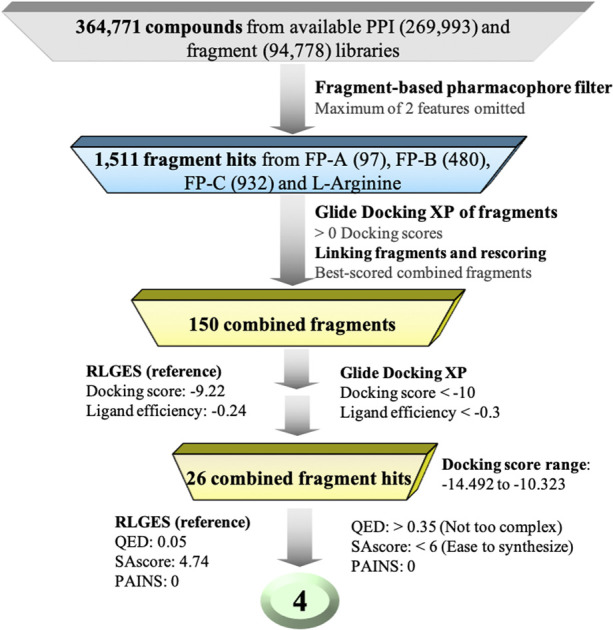

## Introduction

**Fig. 1:**
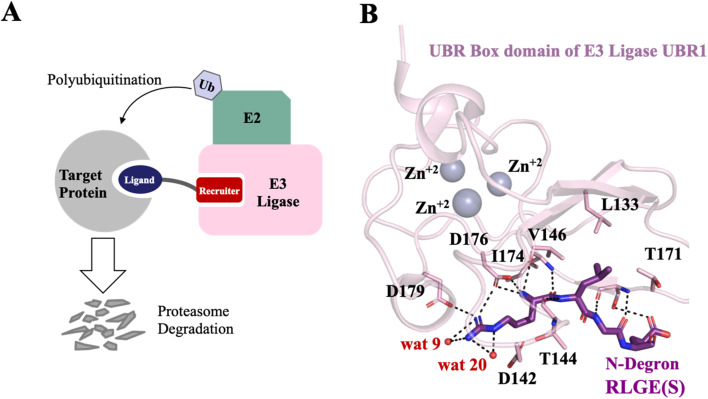
Overview of PROTAC-induced degradation and the UBR1–N-degron interaction. (A) A schematic representation of a PROTAC, consisting of a protein–target ligand and an E3 ligase recruiter connected by a linker, which promotes polyubiquitination and proteasomal degradation of the target protein. (B) Structure of the RLGES N-degron peptide (recruiter moiety) bound to the UBR box domain of E3 Ligase UBR1 (PDB 3NIN).

Proteolysis Targeting Chimeric Molecules (PROTACs) are heterobifunctional molecules that induce the approach of a target protein to the cellular degradation machine.^1–3^ They consist of three essential components: a ligand that binds to the target protein, a recruiter that binds to the E3 ligase, and a linker that connects these two elements. When introduced into the cellular environment, PROTACs form a ternary complex involving the target protein and the E3 ligase. This complex triggers the polyubiquitination of the target protein, marking it for subsequent degradation by the proteasome (Fig. 1A).

The degradation of disease-relevant proteins is an emerging therapeutic strategy for a wide range of diseases, such as cancer, viral infections, and immune and neurodegenerative disorders.^4–6^ PROTACs have demonstrated significant advantages over classic inhibitors, particularly in selectivity and their ability to overcome drug resistance issues. This can be attributed to their induced-degradation mode of action. Once the target protein is marked for proteasomal degradation, PROTACs can dissociate and be recycled for subsequent degradation events. Therefore, PROTACs do not rely on high binding affinities to exhibit potent degradation activities. In contrast, classical small-molecule inhibitors operate through competitive and occupancy-based mechanisms, requiring deep, well-defined binding pockets and high binding affinities. This makes classical inhibitors more susceptible to resistant mutations and target expression increases.^4,7^ The initial step in designing a PROTAC involves the identification of the target protein and an appropriate E3 ligase. PROTACs can be designed to degrade known protein targets by exploiting existing inhibitors as ligands,^8^ but they also offer the potential to explore the “undruggable” proteome that is not susceptible to classic inhibitors.^9^ However, the identification of an efficient cellular degradation machinery is more problematic.^10^ Despite the existence of approximately 600 estimated E3 ligases,^11^ only a few have been utilized, with cereblon and Von Hippel-Lindau being the most commonly employed E3 ligases.^12,13^ The limited repertoire of available cellular degradation machines hampers the development of PROTACS, as they are unable to selectively degrade every protein target, and the expression levels of these E3 ligases are insufficient in certain cell types.^10^ In this context, the N-degron pathway,^14,15^ which is a proteolytic system recognizing N-terminal residues of short-lived proteins through the E3 ligase UBR family, has been recently investigated.^16^

Particularly, UBR proteins are ubiquitously expressed in most cells.^17^ Consequently, PROTACs containing a recruiter for E3 ligase UBR could be exploited to degrade target proteins regardless of the cell type.^18^ In this regard, a rationally designed PROTAC consisting of an N-degron peptide as recruiter linked to a staple peptide as ligand successfully degraded the steroid receptor coactivator-1 (SRC-1), recognized as an oncogenic protein. This study highlights the potential of the N-degron pathway for cancer treatment.^16,19^ However, the clinical progression of PROTACs has encountered obstacles due to their poor cell permeability and proteolytic stability, which is often attributed to their peptide nature and large molecular weight. Furthermore, the large and shallow protein–protein interaction (PPI) surfaces^20–22^ make the design of small molecules with efficient E3 ligase recruiting activity a difficult task.

To overcome such obstacles, we employed an *in silico* peptidomimetics strategy, which involved the virtual screening of compound libraries to identify small molecules that mimic the structural features of N-degrons responsible for their binding affinity while providing higher stability towards proteolysis, better transport properties, and selectivity.^20,23^ Fortunately, crystal structures of N-degron peptides in complexes with UBR1 have been described.^24^ N-degron peptides bind in a relatively shallow acidic cleft located at the UBR box domain. Structural inspection and binding affinity experiments indicated that the first residue (positively charged) of N-degron makes a major contribution to the binding affinity. This is evidenced by the co-crystalized structure of the wild-type peptide (RLGES) from *S. cerevisiae* cohesion subunit Scc1 (PDB 3NIN,^24^Fig. 1B). The guanidinium group of arginine forms an extensive hydrogen-bonding network with the carboxyl groups of D176 and D179, as well as a water-mediated contact with D142. Recognition of its terminal NH_3_^+^ group appears particularly important, as it establishes hydrogen bonds with both the D176 carboxyl group and the backbone of I174. Consistent with its central structural role, D176 interacts with both the guanidinium group and the terminal NH_3_^+^ of arginine. Mutagenesis studies have confirmed that D176 is essential for the UBR box function.^25^ At the second position, the leucine side chain does not completely fill the shallow hydrophobic pocket formed by V146, L133, and T171, suggesting a less important contribution to binding affinity. The third residue, glycine, forms a hydrogen bond with the backbone atoms of S174, while the fourth residue, glutamate, likely plays a minor role since it undergoes deamidation or arginylation prior to recognition by the UBR domain.^24^

Our focus was on designing E3 ligase UBR1 recruiters, aiming to identify a potential set of compounds that could be linked to protein target ligands and effectively degrade disease-relevant proteins in any cell type. Specifically, we developed a fragment-based computational strategy that combined pharmacophore filtering, docking, and fragment-linking optimization. Subsequently, the resulting combined fragments were filtered as a function of docking score, drug-likeness, and synthetic accessibility. Finally, the combined fragments exhibiting the best scores were further evaluated through advanced binding free energy calculations, which revealed the molecular basis of the binding mechanism and the potential of these designed compounds as effective E3 ligase UBR1 recruiters.

## Results and discussion

### Fragment pharmacophore-based virtual screening of the N-degron–UBR box complex

Structure-based virtual screening (SBVS) is a well-established and successful computational methodology that exploits the three-dimensional knowledge of the protein target to select drug candidates from large compound databases.^26^

**Fig. 2:**
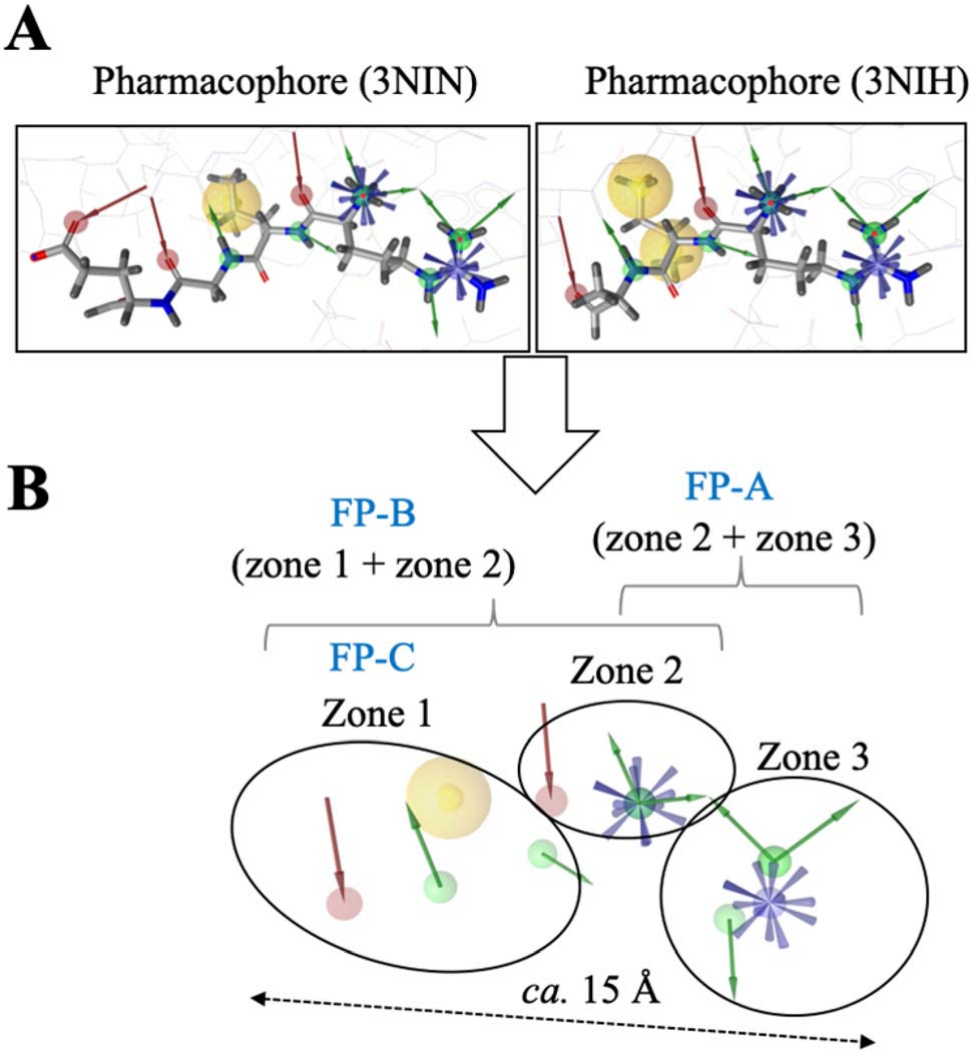
Fragment-based pharmacophore model of UBR1–Ndegron recognition. (A) Pharmacophore features derived from the crystal structures PDB 3NIN and 3NIH. The models include positive ionizable areas (in blue), hydrogen bond donors (in green), hydrogen bond acceptors (in red), and hydrophobic interactions (in yellow). (B) Combined fragment-based pharmacophore model generated from the shared features of 3NIN and 3NIH, comprising three fragment pharmacophores: FP-A, FP-B, and FP-C.

Guided by the available structural information and the reported binding affinity data, which show that arginine is essential as a first residue followed by a hydrophobic residue at the second position (Fig. 1B and Table S1), we constructed the pharmacophore model based on the shared features of the wild-type RLGES and RIAAA peptides, thereby capturing the key determinants of UBR1–N-degron recognition. It is worth noting that the C-terminal residues—serine in RLGES and alanine–alanine in RIAAA—were not resolved in the crystal structures and were therefore excluded from the pharmacophore model. Notably, the resulting pharmacophore closely resembled that of 3NIN, except for the absence of the hydrogen-bond acceptor contributed by the glutamate side chain (Fig. 2A).

The model primarily consisted of positive ionizable areas and hydrogen bond donors. It also included a hydrophobic area and some hydrogen bond acceptors (Fig. 2A). Given that the N-degron and UBR box interface involves protein–protein interactions (PPIs), we started screening PPI commercial libraries. However, there were no compounds that matched the physicochemical properties of the pharmacophore model generated based on the N-degron/UBR box interface. This is because this specific case of PPI produces a pharmacophore with challenging physicochemical properties, including a long length (*ca.* 15 Å) that is hard to target. To overcome this challenge, we decided to develop a fragment-based pharmacophore (FP) model.^27^ Specifically, we divided the original pharmacophore into two overlapping areas, which led to the definition of two fragment pharmacophore models: FP-A (zone 2 + zone 3) and FP-B (zone 1 + zone 2). To further increase the diversity of combinations, the largest zone 1 was also considered independently as FP-C, see Fig. 2B. Subsequently, we performed the screening of fragment libraries using the three fragment pharmacophore models to find compounds that bind to each zone. Fragment libraries are composed of small libraries of low-complexity compounds, which have been proven to present high hit rates. In total, considering the screening of four commercial libraries, we obtained 1511 fragment hits, from which 97 were identified in the FP-A screening, 480 in the FP-B, and 932 in the FP-C (Table S2). Notably, FP-A presents the lowest hit rate, highlighting the structural complexity of zone 3, which makes it more challenging to target than zone 1. However, as described previously, zone 3 is the most critical to the binding energy. The resulting fragment hits are expected to be complementary to the UBR box binding site at their respective binding zones. Nevertheless, to obtain compounds with good binding affinities, these fragments must be further optimized through expansion strategies, such as fragment growing, merging, and linking. This is an attractive approach to discovering new chemical entities with high specificity.^28^

### Docking of fragment hits in the UBR1binding site followed by linking optimization

Molecular docking is an efficient method for ranking a large number of compounds based on the estimation of binding affinity.^29,30^ More importantly, it offers a 3D visualization of the virtual hits docked in the binding pocket. This structural information aids in the execution of hit optimization procedures. In this work, a fragment linking optimization protocol as described in the “combine fragment” panel of Schrodinger software was used. First, the 1511 fragment hits were docked in the UBR1 binding site using Glide Docking, and second, the fragments that were docked in proximity were joined by identifying bonds that can be formed according to geometrical criteria. For fragment pairs that have no atoms close enough to create new bonds but still not far apart, at most two methylene linkers can be included. Next, the resulting combined fragments were docked, and the 150 top-scoring 150 were selected for subsequent analysis. In this study, the RLGES peptide is used as a reference recruiter, as it is a well-characterized recruiter of UBR1 derived from the *S. cerevisiae* cohesion subunit Scc1, and targeted for degradation during the metaphase–anaphase transition.^24^ Additionally, an RLGES-based analogue (RLAA) has been successfully employed as a recruiter in PROTAC design to degrade oncogenic targets.^16^

Considering the docking score and the ligand efficiency (docking score/number of heavy atoms) of the RLGES peptide reference values (docking score: −9.22 kcal mol^−1^ and ligand efficiency: 0.24 kcal per mol per heavy atom), we established threshold values of −10 kcal mol^−1^ for the docking score and −0.3 kcal per mol per heavy atom for the ligand efficiency, resulting in 26 combined fragments, all of them exhibiting superior docking score and ligand efficiency compared to the RLGES peptide.

The docking was performed using a recently solved cryo-EM structure of E3 ligase UBR1, which includes the complete UBR1 structure in complex with ubiquitin-conjugating enzyme (ubc2), ubiquitin (Ub), and N-degron (PDB 7MEX).^31^ Based on this structure, we also considered the broader protein surroundings of the UBR box, promoting complementarity with the docked combined hits. Indeed, we observed that docking poses generated using only the UBR box structure can extend into regions that are occupied by other regions of UBR1, leading to potential steric clashes. As expected, the combined fragments obtained in this study are complementary to both the UBR box and its surroundings, yielding a better fit in the UBR1 binding interface (Fig. S1). These findings suggest that incorporating the complete protein context of the UBR1 binding site during the virtual screening process is critical for the design of efficient E3 ligase UBR1 recruiters.

### Safety and molecular properties of the resulting combined fragments

The designed combined fragments were generated through an *in silico* process where low-complexity compounds were joined through methyl or methylene linkers. Therefore, it is crucial to evaluate the safety and the synthetic accessibility of these compounds before considering their application as E3 ligase recruiters. To that end, we evaluated their ADME-tox profiles using the QikProP^32^ module in Maestro. We further assessed drug-likeness using the quantitative estimation of drug-likeness (QED),^33^ and screened for Pan Assay Interference Compounds (PAINS),^34^ while estimating the synthetic accessibility (SA) score,^35^ all calculated using RDKit Python functions.^36^ The ADME-Tox profile was evaluated based on the number of calculated properties that fall outside the range typically observed for ∼95% of known oral drugs, as reported by QikProp. A lower number of out-of-range properties indicates a higher likelihood of favorable pharmacokinetic behavior. The quantitative estimation of drug-likeness (QED) ranges from 0 (all properties unfavorable) to 1 (all properties favorable). The synthetic accessibility (SA) score ranges from 1 (easiest to synthesize) to 10 (most difficult to synthesize), and ideally, no pan-assay interference compounds (PAINS) should be present.

We started evaluating the RLGES peptide, which, as expected, presents a significantly low drug-likeness (QED: 0.04). However, it is easy to synthesize (SA score: 4.9) and does not contain any PAINS compounds. Subsequently, the evaluation of the 26 combined fragments reveals a substantial enhancement in drug-likeness compared to the RLGES peptide (QED: 0.09–0.48). These combined fragments are also feasible to synthesize (SA score: 4.33–6.67), are free from PAINS compounds, and present a relatively low number of out-of-range ADME-Tox properties (0–10). These molecular descriptors provide an overall estimation of the suitability of the combined fragments and can also be used as an additional filter to prioritize fragment candidates. Considering that compounds with fewer than five out-of-range ADME-Tox properties are generally regarded as acceptable candidates,^37^ QED values above 0.35 indicate acceptable drug-likeness,^33^ and SA scores above 6 are typically difficult to synthesize,^35^ we applied these thresholds to filter and obtain a refined set of combined fragments. This resulted in 4 best-scoring combined fragments, named BCF1–4, which present a favorable safety profile, see Table S3.

**Fig. 3:**
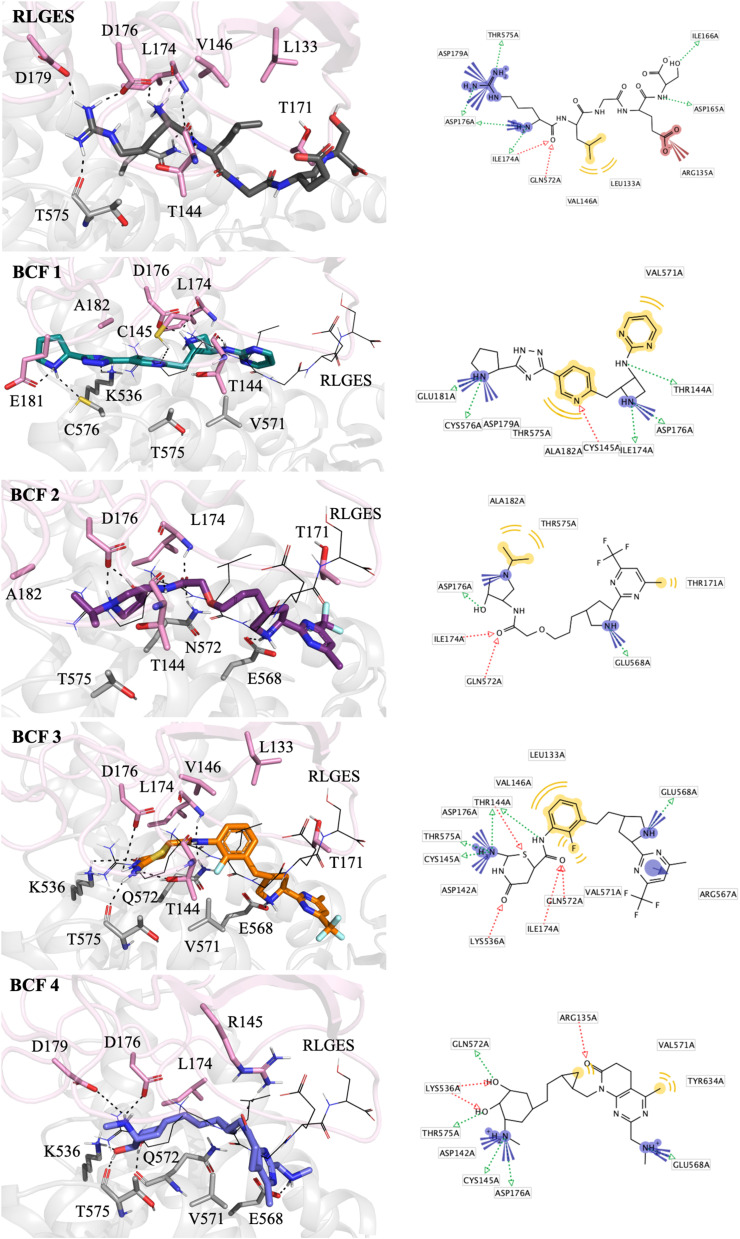
Representation of docking poses and pharmacophore features of the RLGES peptide and BCF 1–4. The UBR box is colored pink while the protein surroundings are grey. In the right panels, ligands and interacting residues are shown as sticks, with polar contacts depicted as dashed black lines. For the BCFs, the docking pose of RLGES is superimposed and shown as black lines for comparison. In the left panels, the pharmacophore features of all ligands are displayed. The models highlight positive ionizable areas (in blue), hydrogen bond donors (in green), hydrogen bond acceptors (in red), and hydrophobic interactions (in yellow).

Analysis of the docking structures demonstrated that both the reference peptide RLGES and the best combined fragments (BCFs) reproduced key pharmacophore features and established interactions with major residues at the UBR1 binding interface (I174, D179, D176, I174, V146, L133, T171, T144, and D142), as well as with surrounding residues of the UBR box (Q572, K536, T575, and E568) (Fig. 3). As expected, the docking pose of RLGES in UBR1 closely resembles both the pharmacophore model and the experimental RLGE–UBR box crystal structure (Fig. 3 and S2A). The arginine residue forms polar contacts with D179, D176, I174, and T144 while also interacting with T575 and N572 from the protein surroundings. Meanwhile, the leucine residue establishes hydrophobic interactions with V146, L133, and T171.

Superimposition of the RLGES docking pose with those of the BCFs (see RLGES in black lines, Fig. 3) shows partial spatial overlap, indicating that the BCFs reproduce several key pharmacophore interactions while adopting distinct orientations within the binding pocket. The BCFs often retain the main anchoring contacts observed for RLGES—particularly with D176, I174, and T144—while forming new interactions with neighboring residues within the same binding region, reflecting their distinct chemical composition. This indicates compatibility between functional moieties of the BCFs and the local chemical environment of the binding pocket. For example, BCF1 extends deeper into the binding site, establishing additional interactions with E181 and C576 while preserving key contacts with D176, I174, and T144. In contrast, BCF2, the polar interaction of arginine with D179 is replaced by hydrophobic contacts involving its isopropyl group and residues A182 and T575, while maintaining relevant interactions with D176, T144, I174, and T171, as well as nearby residues such as N572. Similarly, BCF3 preserves the hydrophobic interactions observed for RLGES with residues V146 and L133. These observations confirm that the BCFs partially follow the pharmacophore model, maintaining the essential binding determinants of the N-degron–UBR1 recognition while exploring complementary regions of the binding interface.

Interestingly, all the designed recruiters exhibit solvent exposure areas, which highlights their potential to be linked to target protein ligands without affecting their binding affinity. This manageable number of compounds is suitable for a more comprehensive computational evaluation and investigation of their potential as UBR1 E3 ligase recruiters.

### Evaluation of the binding free energy surface (BFES) of the best-scored combined fragments

The reconstruction of the BFES is essential to evaluate the performance of the designed recruiters. It informs about the relative stability of the recruiter in the UBR1 E3 ligase binding site and provides insights into the binding pathway and mechanism. Nonetheless, the reconstruction of the BFES is often challenging due to the long timescale of the binding process, requiring the use of advanced molecular dynamics simulation techniques.^38^ To provide a preliminary assessment of the bound-state stability, we conducted conventional molecular dynamics simulations of the RLGES peptide and the BCF1–4 compounds, using the docking poses as starting structures.

The ligand RMSD and distances to the key anchoring active-site residues D176, I174, and T144 indicate that the reference ligand and the BCF compounds remain in the bound-state conformation, with mean RMSD values in the 2–4 Å range, consistent with modest to moderate structural rearrangements relative to their docking poses (Tables S4 and S5).

Given that, all the compounds were considered for the reconstruction of the binding pathway. To that end, we employed funnel metadynamics (FM) simulation, a recently enhanced sampling technique that has demonstrated great efficiency in computing the BFES for a variety of protein–ligand systems.^39–42^ FM accelerates the BFES reconstruction by applying a bias potential that prevents the trapping of the system in a stable protein-bound conformation by applying a funnel-shaped restraint potential to restrict the ligand exploration to the region of interest, avoiding the sampling of the ligand around the entire bulk solvent. The funnel-shaped restraint consists of a conic region that encompasses the protein binding site connected to a cylindrical region directed towards the solvent (see more details in Fig. S3).

**Fig. 4:**
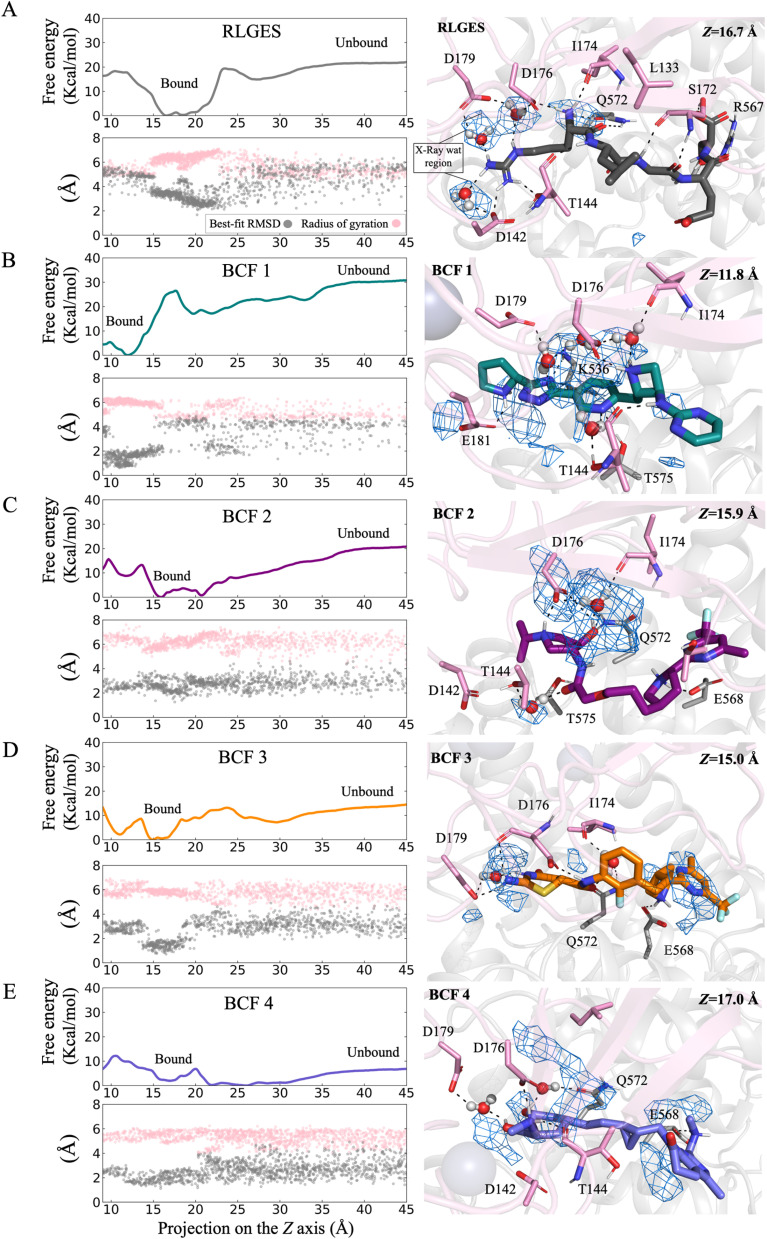
Binding Free Energy Surface (BFES) of RLGES peptide and BCF1–4. The BFESs were reconstructed from FM simulations as a function of the progression of the ligand center of mass (COM) along the *Z* axis of the funnel: RLGES (A) and BCF1–4 (B)–(E). Below each BFES, the best-fit RMSD using the docking poses as reference (gray dots) and the radius of gyration (pink dots) are plotted as a function of the *Z* axis. The right panels show representative conformations of the bound states. The UBR box is colored pink, while the protein surroundings are grey. Ligands and interacting residues are shown as sticks. Polar interactions between ligands, water molecules, and protein residues are indicated as black dashed lines. Regions of high-water density, calculated using the VolMap tool in VMD, are displayed as a blue mesh.

**Fig. 5:**
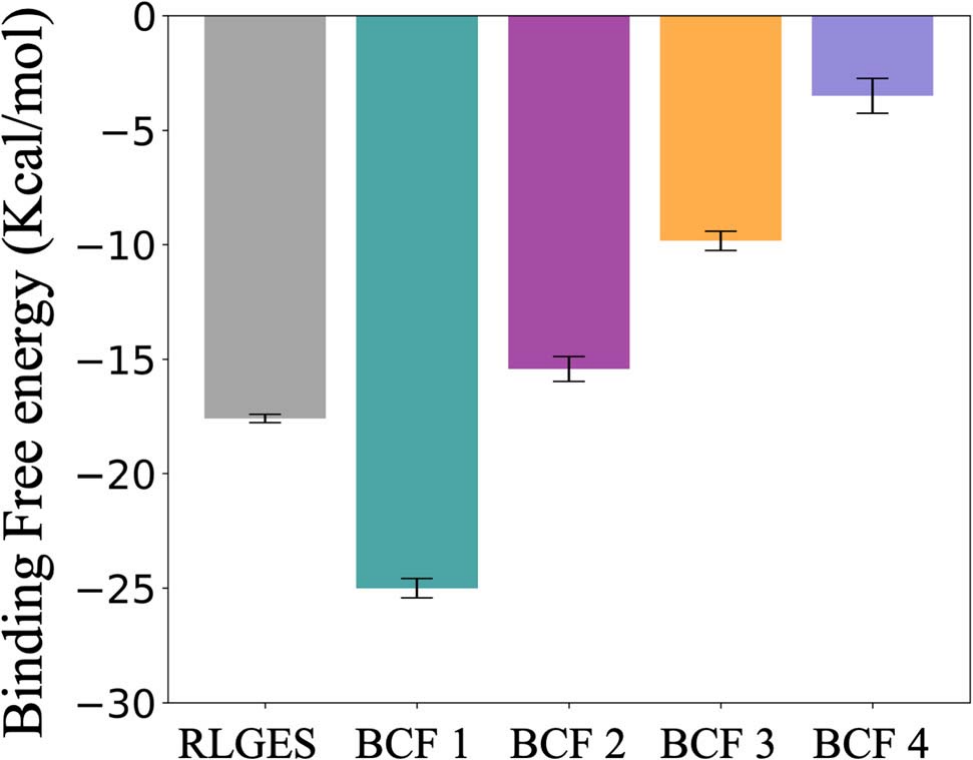
Binding free energy estimates of the RLGES peptide and BCF 1–4. These values were calculated as the average ΔΔ*G* between ligand-bound and unbound regions over the last 200 ns of the simulation (see Materials and methods section in the SI and Fig. S12), with their corresponding uncertainties estimated as the standard deviation from these average values.

We started by computing the BFES of the RLGES wild-type peptide (Fig. 4A and 5). The BFES of the RLGES peptide showed that the bound state is highly stable. As observed experimentally, the bound conformations exhibit a robust hydrogen bond network. This network comprises water-mediated and direct hydrogen bonds connecting the guanidinium group of RLGES and residues D179, D176, D142, and T144, and the NH_3_^+^ group of RLGES and residues D176, I174, and Q572 (Fig. 4A and Movie S1). It is worth noting that individual waters are dynamically exchanged during the simulations, which may facilitate conformational rearrangements. This behavior is observed in the guanidine group, which rotates and samples different poses, giving rise to a dynamic water-mediated hydrogen bond network. To further explore the role of water molecules, we computed water density maps during the FM simulations. The structural relevance of these water molecules in mediating ligand-binding site interactions is highlighted by regions of high-water density (blue mesh in Fig. 4A and Movie S1), which include the positions of the crystallographic waters wat9 and wat20 identified in the 3NIN PDB structure, underscoring the importance of these waters for RLGES binding.

At the C-terminal extreme of RLGES, the Ser residue forms hydrogen bonds with residues R567 and S172 while the Glu residue remains solvent-exposed, thus exhibiting higher flexibility. The transient breakage of these hydrogen bonds increases flexibility in the C-terminal region, destabilizing the bound state (Fig. S4). Eventually, the rearrangement of water-mediated interactions involving the guanidine group, along with the loss of key peptide–UBR1 hydrogen bonds, such as the NH_3_^+^ with I174, D176, and Q572, leads to higher energy intermediate conformations (Fig. S5 and Movie S1). At this point, the peptide rapidly evolves towards total unbinding conformations. Upon unbinding, the peptide mostly samples folded conformations in which the guanidine group of Arg and the carboxylic acid group of Ser approach each other, forming a stable salt bridge (Fig. S6 and Movie S1). Ligand folding was monitored using the best-fit RMSD relative to the docking pose and the ligand radius of gyration. The bound-state region is characterized by lower best-fit RMSD and higher radius of gyration values, indicating that the peptide remains in unfolded conformations. Upon unbinding, RLGES folding is reflected by an increase in best-fit RMSD, accompanied by a decrease in the peptide radius of gyration (Fig. 4A).

As discussed above, the RLGES wild-type peptide serves as a reference to evaluate the designed compounds. Our goal is to identify compounds exhibiting BFES profiles similar to the reference peptide. Given the induced-degradation mode of action of PROTACs, efficient degraders require a balance between sufficient binding affinity to enable productive ternary complex formation and adequate dissociation kinetics to allow multiple rounds of target ubiquitination. Compounds with binding free energy much lower than RLGES may form overly stable complexes with UBR1, thereby reducing dissociation rates and limiting recruiter recycling. Conversely, compounds with much higher binding free energies may fail to bind UBR1 efficiently and therefore be inefficient degraders.

The BFES profiles of the designed compounds reveal their potential as recruiters (Fig. 4B–E and 5). BCF1 exhibits a significantly lower binding free energy to RLGES (ΔΔ*G ca.* −7.4 kcal mol^−1^). This suggests that the unbinding process may be too slow and infrequent to serve as an efficient recruiter. In contrast, BCF2 showed a similar profile to that of the RLGES peptide, with a marginal ΔΔ*G* increase of *ca.* 2.2 kcal mol^−1^. BCF3 compound shares the fragment containing the trifluoromethyl group with BCF2. Nevertheless, the simulations indicate that it anchors less effectively through the other fragment, resulting in a ΔΔG *ca.* 5.9 kcal mol^−1^ higher than BCF2 (Fig. 5). Lastly, the BFES for BCF4 presents a very low binding free energy, suggesting that it is expected to be an inefficient recruiter.

As observed for the reference ligand, FM simulations show that the BCFs exhibit high-density regions of water molecules within the binding site. These structural waters mediate ligand binding, and their dynamic interactions facilitates ligand rearrangements, as illustrated by representative bound conformations (Fig. 4). Population analysis of key polar interactions between the reference and designed ligands with anchoring residues of the acidic binding site region – including water-mediated interactions – along the *Z* axis projection, reveals well-defined, high-probability basins in the bound-state region. These results indicate that such polar contacts are stable in the bound state, whereas their disruption promotes ligand unbinding (Fig. S7–S11 and Movies S1, S2).

Consistent with docking predictions, BCF1 forms polar contacts with E181, D176, T144, and R536. FM simulations further reveal additional water-mediated interactions involving D179, I174, and T144 (Fig. 4B). BCF2 is primarily anchored in the active site by polar interactions with D176, I174, Q172, T144, and E568. FM reveals ligand rearrangements that give rise to new water-mediated interactions with D176, T144, and I174 (Fig. 4C and Movie S2). Notably, the trifluoromethyl group can be accommodated in the interface between the UBR box and protein surroundings, where it can make a substantial contribution to the binding energy. In contrast, the methylene group is more solvent-exposed, suggesting it as a more suitable attachment point for linking protein degradation ligands.

Considering the conformational changes of BCFs during unbinding, BCF1 exhibits best-fit RMSD and radius of gyration profiles similar to those of the RLGES peptide. Its pyrimidine and pyridine rings engage in pi–pi stacking interactions, resulting in stable folded conformations upon unbinding (Fig. S6). In contrast, the remaining BCFs display a higher propensity to remain unfolded, as indicated by lower best-fit RMSD and higher radius of gyration values in the unbinding region. This behavior follows the trend BCF2 > BCF3 > BCF4 (Fig. 4B–E and S6). Taken together, these observations indicate that BCF2 presents the most suitable scaffold for recruiting E3 ligase UBR1.

## Conclusions

Proteolysis targeting chimeric molecules (PROTACs) hold promising potential in drug discovery by facilitating the specific degradation of disease-related proteins within cells. However, the clinical progression of PROTACs has faced significant obstacles, mainly attributed to their peptide-based structure and the scarcity of appropriate E3 ligases. Here, we concentrate on creating agents for recruiting the E3 ligase UBR1. This ubiquitously expressed protein has been recently shown to be highly effective in the degradation of oncogenic proteins. Through a fragment-based virtual screening strategy, we have identified fragments that exhibit characteristics resembling the various zones of the wild-type RLGES peptide when it is bound to the UBR1 E3 ligase. Subsequently, an optimization process involving fragment-linking led to the creation of combined fragments. To verify their suitability as therapeutics, we assessed their safety and synthetic accessibility. These combined fragments are expected to mimic the physicochemical properties of the wild-type peptide while overcoming the disadvantages of the peptide, such as stability towards proteolysis and transport properties. The reconstruction of the binding free energy surface (BFES) of the best-scored combined fragments showed their potential utility as recruiters. BCF2 exhibits a similar BFES profile to that of the wild-type recruiter RLGES, making it the most promising design. The BFES calculations also revealed the binding mechanism of both the RLGES peptide and the designed compounds. In all cases, we observed that a dynamic network of water-mediated hydrogen bonds was crucial for stabilizing the bound state. The collection of this dynamic conformational ensemble proves to be valuable for guiding hit-to-lead optimization. Furthermore, upon closer examination of these structures, it is evident that the designed UBR1 E3 ligase recruiter, BCF2, can easily be linked to protein target ligands *via* its solvent-exposed methylene group, thereby enabling the development of efficient PROTACs. Utilizing the UBR1 E3 ligase as a degradation machinery represents a novel approach that holds promise for degrading disease-relevant proteins independent of the cell type. Notably, the fragment-based peptidomimetics strategy combined with the advanced binding free energy calculations devised in this study can also be employed for modeling related PROTACs that entail intricate protein–protein interactions.

## Author contributions

Dr Miguel A. Maria Solano: data curation, formal analysis, conceptualization, investigation, visualization, writing – original draft, writing – review and editing. Prof. Dr Sun Choi: supervision, writing – review and editing, funding acquisition, and resources. Dr Raudah Lazim: data curation, investigation, writing – review and editing. All authors have given approval to the final version of the manuscript.

## Conflicts of interest

The authors declare no competing financial interest.

## Supplementary Material

RA-016-D5RA04908C-s001

RA-016-D5RA04908C-s002

RA-016-D5RA04908C-s003

## Data Availability

To facilitate the reproduction of the results, we have made all ligand-bound initial configurations, topology files, and an example of a PLUMED input file, which includes the parameters and conditions used for the FM-simulations, available on GitHub (https://github.com/biochem0904/UBR1-Ndegron). Additionally, to enable users to customize their analysis, the repository also includes bound-state representative conformations derived from the FM-simulations. The simulation trajectories are available from the corresponding author upon reasonable request. Supplementary information (SI): materials and methods, tables, figures, and movies. See DOI: https://doi.org/10.1039/d5ra04908c

## References

[cit1] Burslem G. M., Crews C. M. (2020). Proteolysis-Targeting Chimeras as Therapeutics and Tools for Biological Discovery. Cell.

[cit2] Cromm P. M., Crews C. M. (2017). Targeted Protein Degradation: From Chemical Biology to Drug Discovery. Cell Chem. Biol..

[cit3] Bondeson D. P., Mares A., Smith I. E., Ko E., Campos S., Miah A. H., Mulholland K. E., Routly N., Buckley D. L., Gustafson J. L. (2015). *et al.*, Catalytic in vivo protein knockdown by small-molecule PROTACs. Nat. Chem. Biol..

[cit4] Sun X., Gao H., Yang Y., He M., Wu Y., Song Y., Tong Y., Rao Y. (2019). PROTACs: great opportunities for academia and industry. Signal Transduct. Targeted Ther..

[cit5] Nalawansha D. A., Crews C. M. (2020). PROTACs: An Emerging Therapeutic Modality in Precision Medicine. Cell Chem. Biol..

[cit6] Chamberlain P. P., Hamann L. G. (2019). Development of targeted protein degradation therapeutics. Nat. Chem. Biol..

[cit7] Joseph J. D., Lu N., Qian J., Sensintaffar J., Shao G., Brigham D., Moon M., Maneval E. C., Chen I., Darimont B. (2013). *et al.*, A clinically relevant androgen receptor mutation confers resistance to second-generation antiandrogens enzalutamide and ARN-509. Cancer Discov..

[cit8] Winter G. E., Buckley D. L., Paulk J., Roberts J. M., Souza A., Dhe-Paganon S., Bradner J. E. (2015). DRUG DEVELOPMENT. Phthalimide conjugation as a strategy for in vivo target protein degradation. Science.

[cit9] Lai A. C., Crews C. M. (2017). Induced protein degradation: an emerging drug discovery paradigm. Nat. Rev. Drug Discov..

[cit10] Belcher B. P., Ward C. C., Nomura D. K. (2023). Ligandability of E3 Ligases for Targeted Protein Degradation Applications. Biochemistry.

[cit11] Komander D., Rape M. (2012). The ubiquitin code. Annu. Rev. Biochem..

[cit12] Jevtic P., Haakonsen D. L., Rape M. (2021). An E3 ligase guide to the galaxy of small-molecule-induced protein degradation. Cell Chem. Biol..

[cit13] Kannt A., Dikic I. (2021). Expanding the arsenal of E3 ubiquitin ligases for proximity-induced protein degradation. Cell Chem. Biol..

[cit14] Bachmair A., Finley D., Varshavsky A. (1986). In vivo half-life of a protein is a function of its amino-terminal residue. Science.

[cit15] Varshavsky A. (2019). N-degron and C-degron pathways of protein degradation. Proc. Natl. Acad. Sci. U. S. A..

[cit16] Lee Y., Heo J., Jeong H., Hong K. T., Kwon D. H., Shin M. H., Oh M., Sable G. A., Ahn G. O., Lee J. S. (2020). *et al.*, Targeted Degradation of Transcription Coactivator SRC-1 through the N-Degron Pathway. Angew. Chem., Int. Ed..

[cit17] Kwon Y. T., Reiss Y., Fried V. A., Hershko A., Yoon J. K., Gonda D. K., Sangan P., Copeland N. G., Jenkins N. A., Varshavsky A. (1998). The mouse and human genes encoding the recognition component of the N-end rule pathway. Proc. Natl. Acad. Sci. U. S. A..

[cit18] Shanmugasundaram K., Shao P., Chen H., Campos B., McHardy S. F., Luo T. P., Rao H. (2019). A modular PROTAC design for target destruction using a degradation signal based on a single amino acid. J. Biol. Chem..

[cit19] Lee Y., Yoon H., Hwang S. M., Shin M. K., Lee J. H., Oh M., Im S. H., Song J., Lim H. S. (2017). Targeted Inhibition of the NCOA1/STAT6 Protein-Protein Interaction. J. Am. Chem. Soc..

[cit20] Pelay-Gimeno M., Glas A., Koch O., Grossmann T. N. (2015). Structure-Based Design of Inhibitors of Protein-Protein Interactions: Mimicking Peptide Binding Epitopes. Angew Chem. Int. Ed. Engl..

[cit21] Macalino S. J. Y., Basith S., Clavio N. A. B., Chang H., Kang S., Choi S. (2018). Evolution of In Silico Strategies for Protein-Protein Interaction Drug Discovery. Molecules.

[cit22] Qiu Y., Li X., He X., Pu J., Zhang J., Lu S. (2020). Computational methods-guided design of modulators targeting protein-protein interactions (PPIs). Eur. J. Med. Chem..

[cit23] Lenci E., Trabocchi A. (2020). Peptidomimetic toolbox for drug discovery. Chem. Soc. Rev..

[cit24] Choi W. S., Jeong B. C., Joo Y. J., Lee M. R., Kim J., Eck M. J., Song H. K. (2010). Structural basis for the recognition of N-end rule substrates by the UBR box of ubiquitin ligases. Nat. Struct. Mol. Biol..

[cit25] Xia Z., Webster A., Du F., Piatkov K., Ghislain M., Varshavsky A. (2008). Substrate-binding sites of UBR1, the ubiquitin ligase of the N-end rule pathway. J. Biol. Chem..

[cit26] Lee J. W., Maria-Solano M. A., Vu T. N. L., Yoon S., Choi S. (2022). Big data and artificial intelligence (AI) methodologies for computer-aided drug design (CADD). Biochem. Soc. Trans..

[cit27] Deyon-Jung L., Morice C., Chery F., Gay J., Langer T., Frantz M. C., Rozot R., Dalko-Csiba M. (2016). Fragment pharmacophore-based in silico screening: a powerful approach for efficient lead discovery. Medchemcomm.

[cit28] de Souza Neto L. R., Moreira-Filho J. T., Neves B. J., Maidana R., Guimaraes A. C. R., Furnham N., Andrade C. H., Silva Jr F. P. (2020). In silico Strategies to Support Fragment-to-Lead Optimization in Drug Discovery. Front. Chem..

[cit29] Paggi J. M., Pandit A., Dror R. O. (2024). The Art and Science of Molecular Docking. Annu. Rev. Biochem..

[cit30] Irwin J. J., Shoichet B. K. (2016). Docking Screens for Novel Ligands Conferring New Biology. J. Med. Chem..

[cit31] Pan M., Zheng Q. Y., Wang T., Liang L. J., Mao J. X., Zuo C., Ding R. C., Ai H. S., Xie Y., Si D. (2021). *et al.*, Structural insights into Ubr1-mediated N-degron polyubiquitination. Nature.

[cit32] Ioakimidis L., Thoukydidis L., Mirza A., Naeem S., Reynisson J. (2008). Benchmarking the Reliability of QikProp. Correlation between Experimental and Predicted Values. QSAR Comb. Sci..

[cit33] Bickerton G. R., Paolini G. V., Besnard J., Muresan S., Hopkins A. L. (2012). Quantifying the chemical beauty of drugs. Nat. Chem..

[cit34] Baell J. B., Holloway G. A. (2010). New Substructure Filters for Removal of Pan Assay Interference Compounds (PAINS) from Screening Libraries and for Their Exclusion in Bioassays. J. Med. Chem..

[cit35] Ertl P., Schuffenhauer A. (2009). Estimation of synthetic accessibility score of drug-like molecules based on molecular complexity and fragment contributions. J. Cheminf..

[cit36] RDKit: open-source cheminformatics, https://www.rdkit.org

[cit37] Elsaman T., Awadalla M. K. A., Mohamed M. S., Eltayib E. M., Mohamed M. A. (2025). Identification of Microbial-Based Natural Products as Potential CYP51 Inhibitors for Eumycetoma Treatment: Insights from Molecular Docking, MM-GBSA Calculations, ADMET Analysis, and Molecular Dynamics Simulations. Pharmaceuticals.

[cit38] Limongelli V. (2020). Ligand binding free energy and kinetics calculation in 2020. Wiley Interdiscip. Rev.-Comput. Mol. Sci..

[cit39] Limongelli V., Bonomi M., Parrinello M. (2013). Funnel metadynamics as accurate binding free-energy method. Proc. Natl. Acad. Sci. U. S. A..

[cit40] Saleh N., Ibrahim P., Saladino G., Gervasio F. L., Clark T. (2017). An Efficient Metadynamics-Based Protocol to Model the Binding Affinity and the Transition State Ensemble of G-Protein-Coupled Receptor Ligands. J. Chem. Inf. Model..

[cit41] Moraca F., Amato J., Ortuso F., Artese A., Pagano B., Novellino E., Alcaro S., Parrinello M., Limongelli V. (2017). Ligand binding to telomeric G-quadruplex DNA investigated by funnel-metadynamics simulations. Proc. Natl. Acad. Sci. U. S. A..

[cit42] Raniolo S., Limongelli V. (2020). Ligand binding free-energy calculations with funnel metadynamics. Nat. Protoc..

