# Supplementary material for: In silico design and binding mechanism of UBR1 E3 ligase recruiters
Source: RSC Adv. 2026 May 19;16(29):26659–68. doi: 10.1039/d5ra04908c (PMC13187912; doi:10.1039/d5ra04908c)
Supplement: RA-016-D5RA04908C-s001 [file RA-016-D5RA04908C-s001.pdf]

Supplementary Information (SI) for RSC Advances.
This journal is © The Royal Society of Chemistry 2026

In Silico Design and Binding Mechanism of UBR1 E3 Ligase Recruiters

Miguel A. Maria-Solano,* Raudah Lazim, and Sun Choi*

Global AI Drug Discovery Center, College of Pharmacy and Graduate School of Pharmaceutical Science, Ewha Womans University, 03760 Seoul, Republic of Korea

SUPPLEMENTARY INFORMATION

Table of contents

Materials and methods……………………………………………………………………………………3

Pharmacophore model and virtual screening……………………………………………………………3

Docking virtual screening and fragment-linking optimization………………………………………3

Molecular dynamics simulations…………………………………………………………………………5

Funnel metadynamics (FM) simulations…………………………………………………………………7

Figures and Tables……………………………………………………………………………………10

Table S1……………………………………………………………………………………………………10

Table S2……………………………………………………………………………………………………11

Table S3……………………………………………………………………………………………………12

Table S4……………………………………………………………………………………………………13

Table S5……………………………………………………………………………………………………14

Table S6……………………………………………………………………………………………………15

Figure S1……………………………………………………………………………………………………16

Figure S2……………………………………………………………………………………………………17

Figure S3……………………………………………………………………………………………………18

Figure S4……………………………………………………………………………………………………19

Figure S5……………………………………………………………………………………………………20

Figure S6……………………………………………………………………………………………………21

Figure S7……………………………………………………………………………………………………22

Figure S8……………………………………………………………………………………………………23

Figure S9……………………………………………………………………………………………………24

Figure S10…………………………………………………………………………………………………25

Figure S11…………………………………………………………………………………………………26

Figure S12…………………………………………………………………………………………………27

Figure S13…………………………………………………………………………………………………28

References……………………………………………………………………………………………29

Materials and methods

Pharmacophore model and virtual screening. The pharmacophore model was generated through LigandScout 4.4[1] using crystal structures 3NIN and 3NIH, which contain the UBR box domain of Saccharomyces cerevisiae UBR1 in complex with the N-Degron peptide (RLGES) from cohesion subunit Scc1, and RIAAA, respectively. For simplicity, two polar contacts with water molecules were manually removed. LigandScout uses a pattern recognition-based approach.[1] First, all molecules from compound libraries are transformed into pharmacophores, and second, it screens with pattern recognition the library of pharmacophores against the given pharmacophore model. The results are the best matching pairs for each feature. For all fragment pharmacophore models (FP-A, FP-B, and FP-C), a maximum of two features were omitted during the screening process to increase the hit rates. The commercial fragment libraries screened are Life Chemicals (https://lifechemicals.com/screening-libraries/fragment-libraries), Asinex (https://www.asinex.com/building-blocks/fragments),

Otava (https://otavachemicals.com/products/fragment-libraries/general-fragment-library), and ChemDiv (https://www.chemdiv.com/catalog/complete-list-of-compounds-libraries/). The distribution of the physicochemical properties for the commercial fragment libraries used in this study is provided in Table S6.

Docking virtual screening and fragment-linking optimization. The crystal structures of both the UBR box domain of Saccharomyces cerevisiae UBR1 (PDB 3NIN) and the whole UBR1 length (i.e., UBR box domain + protein surroundings) (PDB 7MEX) were prepared using the protein preparation Wizard module[2] in Schrodinger Suite. It includes filling in missing side chains through Prime,[2] the assignment of protonation states and H-bond optimization through

PROPKA, and an all-protein atom minimization using the OPLS4 force field.[3] The 1,511 fragment hits obtained from the pharmacophore virtual screening were prepared using the LigPrep module[4] in Schrodinger Suite, where for each fragment, all ionizable states significantly populated at pH 7.0 +/- 2.0 were generated through Epik,[5] and all structures were energy minimized using the OPLS4 force field.[3] For the docking calculation, we used the Ligand Docking Glide XP (Extra-Precision) module[6] of Schrodinger Suite. As recommended by Schrodinger for fragment docking, we increased the initial number of poses per ligand from 5,000 to 50,000; widened the scoring window from 100 to 500; increased the number of minimized poses per ligand to 1,000; and used the expanded sampling (https://my.schrodinger.com/support/article/582). Subsequently, we employed the “Combine Fragments” module in the Schrodinger Suite to covalently link the pre-positioned fragments on the UBR1 binding site. This method identifies bonds that can be formed between fragments according to geometric criteria. To ensure a proper rotational alignment of the fragments, the angle between the bond directions was set to be less than 15°. To ensure a proper translational alignment of the fragments, the distance between the atom in one fragment and the atom in the other fragment was set to be less than 1 Å. For fragments that have no atoms close enough to create new bonds but are still not far apart, a methylene linker per fragment can be included to try to link those pairs of fragments. Fragments were joined in one single round, i.e., combined fragments were no further subjected to joining a third fragment. Finally, the resulting combined fragments were ranked, and only the top 150 structures were retrieved.

To validate the selected docking software, we re-docked the co-crystalized RLGES ligand into the UBR1 box binding site. The superimposition of co-crystalized and docking structures shows

a high degree of structural similarity between the experimental and predicted poses (Fig. S2A). To further confirm the reliability of the docking protocol, we compared the co-crystalized structure of the RHG N-degron peptide in the binding site of the full-length UBR1 protein with the docking pose of RLGES. This comparison also revealed a high level of structural agreement, particularly for the first two residues (Fig. S2B).

Molecular dynamics simulations. *System preparation*: The E3 ubiquitin-protein ligase UBR1 (PDB 7MEX) comprises 1,950 residues. Given the extensive size of the protein, we constructed a system that comprises the UBR box domain (residues 115-194; 80 residues + three Zinc coordinating ions) and its protein surroundings (residue 211-284 and 305-645; 415 residues), see Fig. S3. Thus, we manually cut the protein chains and added acetyl (Ace) and N-methylamine (Nme) caps in the C and N-terminal residues, respectively. In total, we constructed 5 systems, the UBR1 in complex with the RLGES wild-type peptide and the best-scoring combined fragments (BCF 1-4). For all ligands, the starting pose was derived from the docking calculations. Force field parameters for the combined fragments (BCF 1-4) were generated with the parmchk module of AMBER20[7] using the general Amber force field (GAFF). The atomic charges were obtained using the AM1-BCC model[8] through the antechamber module of AMBER20.[7] The ff14SB force-field was used for the protein, GAFF2 for the ligand, and TIP3P for the water molecules. The structural Zinc ions were included in all simulations and were treated using a non-bonded model. The corresponding Zn^{2+} parameters were taken from the standard AMBER force-field implementation used in this work. All systems were filled into a pre-equilibrated cubic box with a 25-Angstrom buffer of water molecules and neutralized by the addition of explicit counterions (Na+ and Cl-).

Molecular dynamics protocol: The systems were minimized in a two-stage geometry optimization approach. First, a short minimization was performed under constant volume periodic boundary conditions, allowing only water molecules and counterions to relax, while positional restraints with a force constant of 210 Kcal/mol Å2 were applied to the protein and ligands. Second, an unrestrained minimization including all atoms in the simulation cell was carried out. All systems were heated using seven 50 ps steps, incrementing the temperature by ca. 50 K each step (0-298.15 K). Decreasing harmonic restraints were applied to the protein (with force constants of 210, 165, 125, 85, 45, and 10 Kcal/mol Å2) during the thermal equilibration, with the Langevin thermostat used to equalize and control the temperature. During the heating process, the initial velocities were randomized. For the heating and following steps, bonds involving hydrogen were constrained with the SHAKE algorithm, and the time step was set to 2 fs, allowing potential inhomogeneities to self-adjust. An 11 Å cutoff value was applied to Lennard-Jones and electrostatic interactions. The equilibration step was performed in three stages. In the first stage, an MD simulation of 1 ns under NVT ensemble and periodic boundary conditions was performed to relax the simulation temperature. In the second stage, an MD simulation of 2 ns under an NPT ensemble at a simulation pressure of 1.0 bar was performed to relax the density of the system. In the third stage, an MD simulation of 2 ns under an NVT ensemble was performed to further relax the density/volume defined by pressure. After the equilibration of the system, we performed an MD production run of 100 ns for each system.

Funnel metadynamics (FM) simulations. *FM protocol:* FM is a powerful method to reconstruct the binding free energy surface of protein-ligand complexes as a function of a few user-defined descriptors, also referred to as collective variables (CVs).[9] In this work, we selected the progression of the center of mass (COM) of the ligand along the Z-axis of the funnel (see Figure S3 for visualization and customization parameters of the funnel shape potential). The simulations were carried out using the Amber20 code[7] together with the Plumed-master plugin, which contains the FM module v2.0.[10, 11] During an FM simulation, external energy quantities (Gaussian functions) are added to the CV values along the simulation time. This external potential encourages the system to escape from local energy minima and overcome energy barriers, thus allowing for enhanced sampling of the CV space. After sufficient simulation time, the bias potential converges, and the free energy landscape can be reconstructed by summing the Gaussian functions added to the CV values along the simulation time. Here, Gaussian functions of height 2.0 kJ mol-1 and width 0.01 were deposited every 2 ps of the MD simulation at 300 K. For a smooth convergence of the bias potential, we used the well-tempered (WT) version of the metadynamics algorithm, in which the height of the gaussian potentials were gradually decreased over time proportional to the potential deposited in the currently visited point of the CV space. A bias factor parameter of 20 was selected to control how quickly the Gaussian height is decreased. In addition, the multiple-walkers approach was used to improve the conformational sampling and to speed up the metadynamics simulations. It is based on running in parallel interacting replicas (walkers) where each walker biases the identical CV and reads the Gaussian functions deposited by the others during the simulation, thus reconstructing the same metadynamics bias simultaneously. In particular, we run 5 walker replicas (W1-5) for

each system. The structures used as a starting point were the ligand-bound docking poses after system minimization and equilibration as described in the molecular dynamics protocol. After 1-1.2 μs of accumulated simulation time for each system, we reconstructed their binding free energy surfaces by summing the Gaussian potentials deposited by all walker replicas as a function of the CV.

Convergence assessment: An indicator of convergence is that the BFES remains stable and does not change significantly over time while the system continues exploring bound and unbound conformations. We estimate the convergence of the recovered BFESs by monitoring the free energy difference (ΔΔG) between bound and unbound regions throughout the simulation (Fig. S12). The FM simulations were considered converged once the energy differences between the selected regions became progressively flattened and stabilized over simulation time. During this period, walker replicas simultaneously sampled both bound and unbound conformations (Fig. S13). As shown in Fig. S13, in all systems, several walker replicas escaped from the bound-state energy minima (starting point configuration), collectively exploring the entire conformational space of the binding process. These analyses support the successful reconstruction of the BFES and indicate a reasonable degree of convergence.

Binding free energy estimation: Binding free energies were calculated as the average ΔΔG between the ligand-bound and unbound regions after convergence, using the last 200 ns of the simulations (Figs. 5 and S12). Uncertainties were estimated as the standard deviation from these average values. An entropy correction of ca. 3.3 kcal/mol was applied to account for the funnel shape potential in the unbound state. The entropy correction was calculated using the equations described in Ref. 35 of the manuscript.

Software availability: All funnel metadynamics simulations were performed using the Amber package (freely available for academic use at http://ambermd.org/GetAmber.php and the open-source Plumed-master plugin for MD software (https://github.com/plumed/plumed2). To customize the funnel-shaped restrained potential, we employed the "funnel.tcl" plugin for Visual Molecular Dynamics (VMD) software, which provides a user-friendly graphical user interface (GUI). This plugin is available in the GitHub repository at: https://github.com/limresgrp/FMAP_v1/tree/master/funnel_gui.

Figures and Tables

Table S1. X-Ray and binding affinity data available for UBR1 box – N-Degron peptides.

PDB Code	K_d (μM)	N-Degron peptide
3NIH	---	RIAAA
---	7.30	RIAA
3NII	58.5	KIAA
3NIJ	12.9 (low pH)	HIAA
3NIL	343.2	RDAA
3NIK	358.8	REAA
3NIM	17.7	RRAA
3NIN	12.4	RLGES (Scc1)
---	4.22	RLAA

Data from *Nat. Struct. Mol. Biol.* 2010, 17, 1175 (Ref. 25 in the main manuscript)

Table S2. Summary results of the fragment-based pharmacophore virtual screening.

**Commercial Libraries**	**FP-B hits**	**FP-C hits**	**FP-A hits**
**PPI**<br>(ippdb, Asinex, ChemDiv, Otava and Life Chemicals)	**173**/269,993 (0.06 %)	Not Tested	**30**/269,993 (0.01 %)
**Fragment**<br>(Asinex, ChemDiv, Otava and Life Chemicals)	**340**/94,778 (0.4 %)	**967**/94,778 (1 %)	**72**/94,778 (0.08 %)
**Total**<br>(removing duplicates)	**480**/ 364,771 (0.1 %)	**932**/94,778 (1 %)	**97**/ 364,771 (0.03 %)

The virtual hit numbers are highlighted in bold, and the hit rates are shown in brackets.
In the screening process, a maximum of two pharmacophore features could be omitted.

Table S3. Summary results of ADME-tox, drug likeness, and synthetic accessibility of best-scoring combined fragments.

Combined Fragment	Qik-Prop ADME-Tox (N properties outside the range observed for ~95% of known oral drugs)	Quantitative Estimation of Drug-likeness (QED)	Synthetic Accessibility (SA) score	Pan Assay Interference Compounds (PAINS)
BCF 1	0	0.49	5.7	0
BCF 2	0	0.36	5.5	0
BCF 3	0	0.41	5.1	0
BCF 4	0	0.41	5.6	0

Table S4. Ligand RMSD values obtained from conventional MD simulations, calculated for ligand heavy atoms relative to the docking poses after alignment on the protein.

Ligand	Mean RMSD ± SD (Å)
RLGES	3.73 ± 0.49
BCF1	2.37 ± 0.31
BCF2	3.08 ± 1.55
BCF3	3.56 ± 0.59
BCF4	2.24 ± 0.44

Table S5. Key distances between ligands and binding site residues derived from the docking poses and from the conventional MD simulations.

Ligand	Atom pair distance	Docking pose (Å)	MD ensemble (mean ± SD, Å)
RLGES	(R)LGES_N and D176_CG	3.2	3.23 ± 0.11
	(R)LGES_N and I174_O	2.9	2.80 ± 0.10
	R(L)GES_N and T144_O	3.2	2.95 ± 0.14
BCF1	BCF1_N4 and D176_CG	3.2	3.18 ± 0.12
	BCF1_N4 and I174_O	3.5	3.62 ± 0.34
	BCF1_N3 and IT144_O	2.7	2.90 ± 0.17
BCF2	BCF2_N2 and D176_CG	3.5	4.20 ± 0.34
	BCF2_O1 and I174_N	3.0	3.32 ± 0.48
	BCF2_N1 and T144_O	2.9	2.95 ± 0.30
BCF3	BCF3_N1 and D176_CG	4.1	5.86 ± 1.52
	BCF3_O2 and I174_N	2.9	4.30 ± 1.22
	BCF3_N2 and T144_O	2.8	3.45 ± 0.69
BCF4	BCF4_N1 and D176_CG	3.8	3.11 ± 0.13
	BCF4_C3 and I174_CG1	4.2	5.60 ± 1.15
	BCF4_C21 and T144_O	4.0	5.65 ± 0.61

Table S6. Distribution of the physicochemical properties of four commercial fragment libraries used in this study.

Frag. Library	MolWT	ClogP	TPSA (Å^2)	No. of RotB	No. of HBD	No. of HBA	No. of rings
Life Chemicals	≤ 450	≤ 4.5	< 75	≤ 8	≤ 3	≤ 5	> 0
Asinex*	≤ 300	≤ 5	< 190	≤ 9	≤ 6	≤10	> 0
Otava	< 300	< 3	< 80	≤ 3	≤ 3	≤ 4	> 0
ChemDiv	≤ 300	≤ 3	≤ 80	≤ 3	≤ 5	≤ 7	> 0

*Physicochemical properties of the Asinex fragment library were calculated in-house. Statistical data for the other libraries were obtained from their respective official websites.

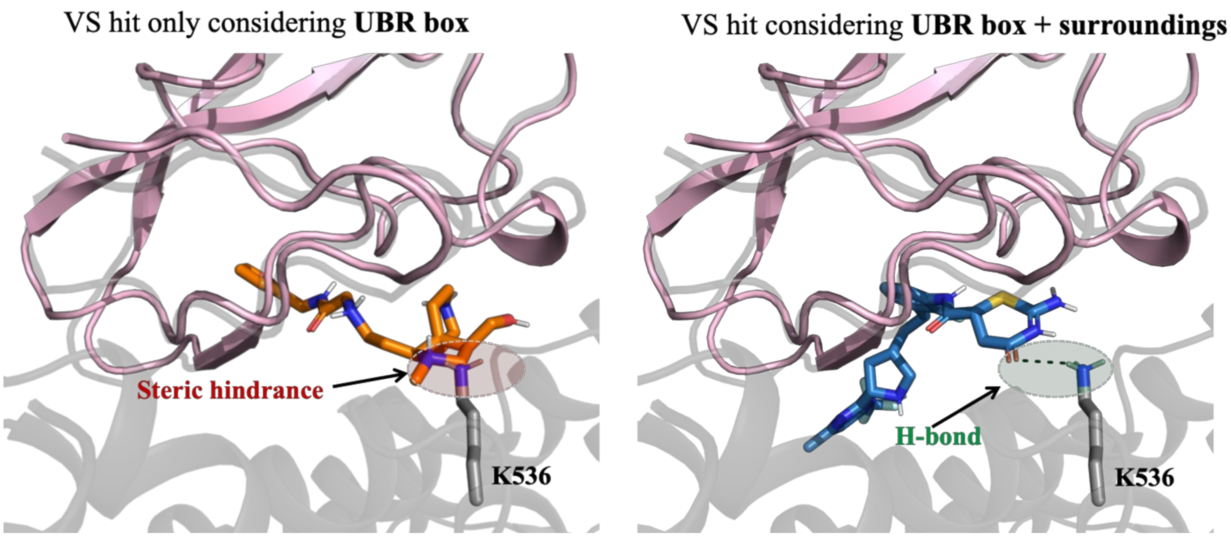

Fig. S1 Representative combined fragments docked in UBR1. Overlap of the UBR box domain (PDB 3NIN), in pink, and the complete length chain of E3 ligase UBR1 (PDB 7MEX), in grey. The docked combined fragment (orange sticks), only considering the UBR box domain in the docking process, performs steric clashes with the UBR box protein surroundings, while the docked combined fragment (blue sticks), considering the complete UBR1 protein, performs favorable interactions.

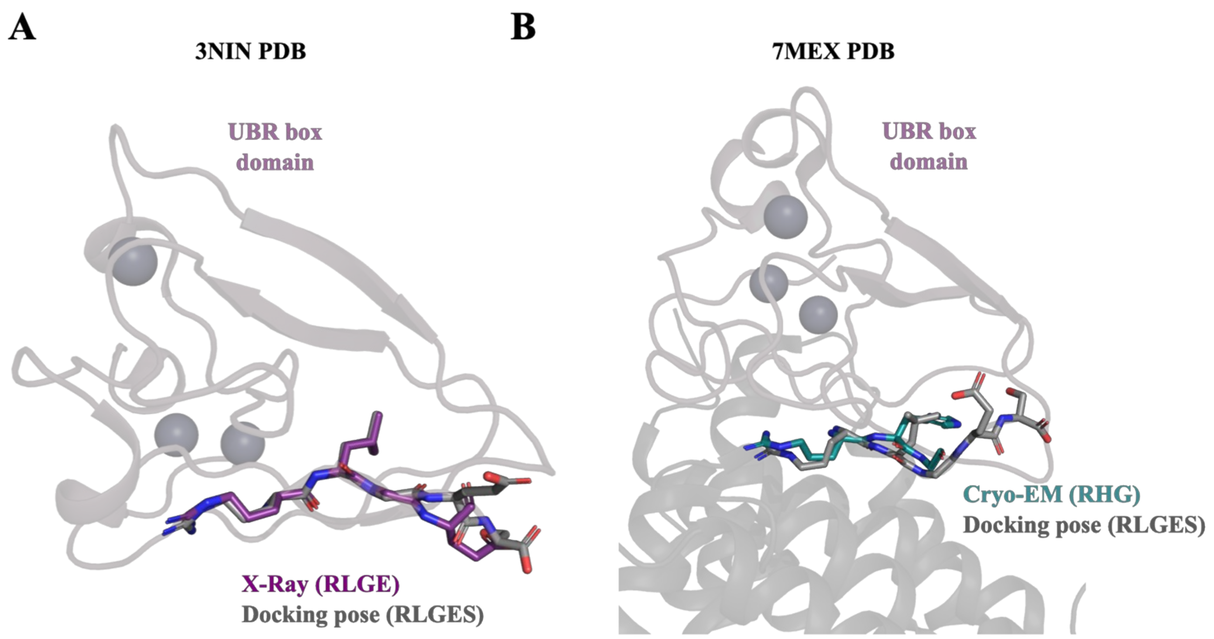

Fig. S2 Comparison of co-crystalized and docking poses of N-degron ligands. **(A)** Superposition of the X-ray structure (purple) and the best-scoring docking pose of the RLGE peptide (gray) in the UBR box domain (PDB 3NIN). **(B)** Superposition of the cryo-EM structure of the RHG peptide (in teal) co-crystalized in the full-length structure of UBR1 (PDB 7MEX) and the best-scoring docking pose of RLGE (in gray).

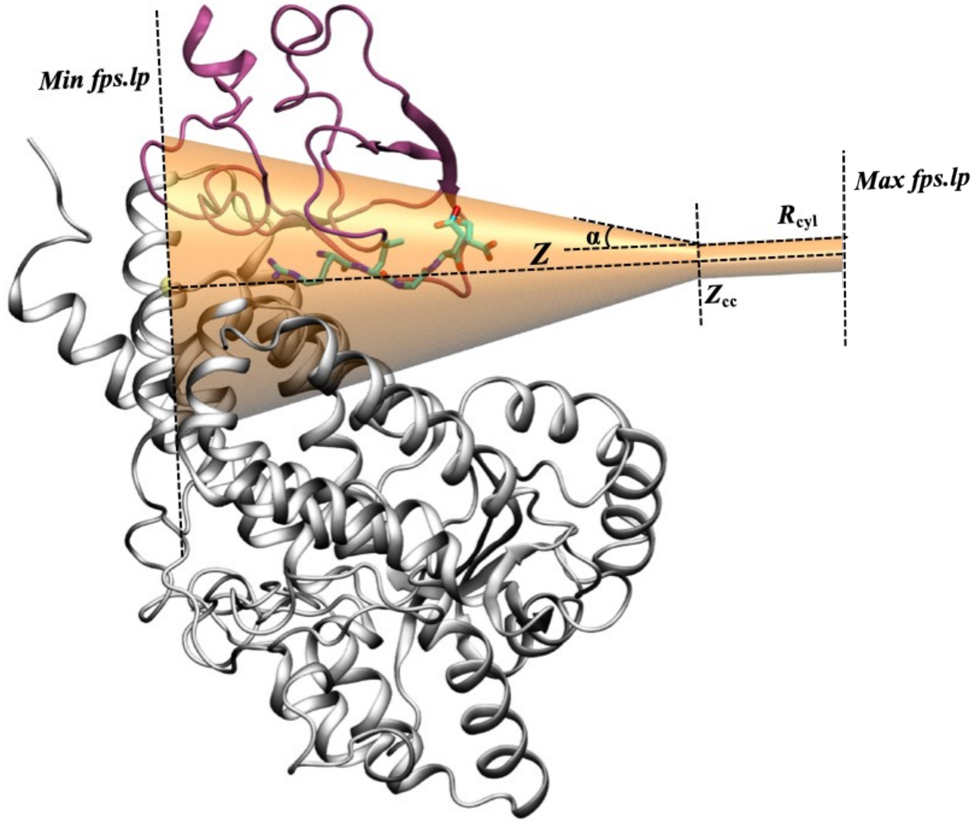

Fig. S3 Representation of the UBR1 protein region and the funnel-shaped restraint potential used in this work. The UBR1 box domain (82 residues, in pink) and the UBR1 protein surroundings (419 residues, in grey) are shown in cartoon style while the RLGES wild-type peptide is in cyan sticks. The conic and cylindrical regions of the funnel restrain potential are depicted in orange. *Z* defines the axis along which the COM of the ligands progresses along the binding pathway. The funnel shape was customized as follows: the minimum and maximum values for fps.lp used to construct the funnel were set to 2 and 52 Å, respectively, giving rise to a funnel length of 50 Å. The point where the funnel shape switches from a cone into a cylinder (Z_{cc}) was set to 45 Å, the angle defining the amplitude of the cone (α) to 0.25 rad, and the radius of the cylindrical region (Z_{cyl}) to 1.5 Å.

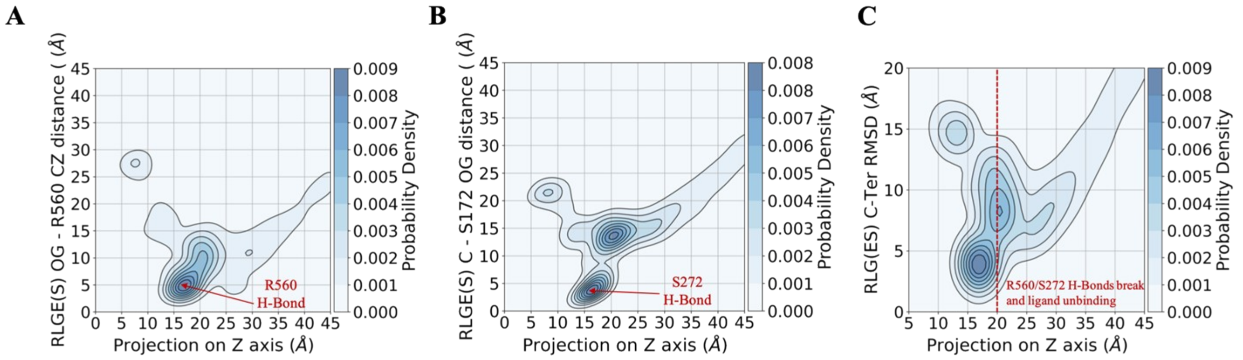

Fig. S4 Population analysis of C-Terminal dynamics of RLGES during unbinding. 2D kernel density plots of **(A)** the distance between the OG atom of RLGE(S) and the CZ atom of R560 along the Z axis, **(B)** the distance between the C atom of RLGE(S) and the OG atom of S172 along the Z axis. The high-probability basin associated with the formation of these H-bonds in the bound region (approximately Z = 16-20 Å) is highlighted. **(C)** 2D kernel density plot of the RMSD of the RLG(ES) backbone atoms along the Z axis, illustrating the correlation between disruption of the R560/S272 H-bonds (represented as a dashed line) and C-Ter ligand displacement.

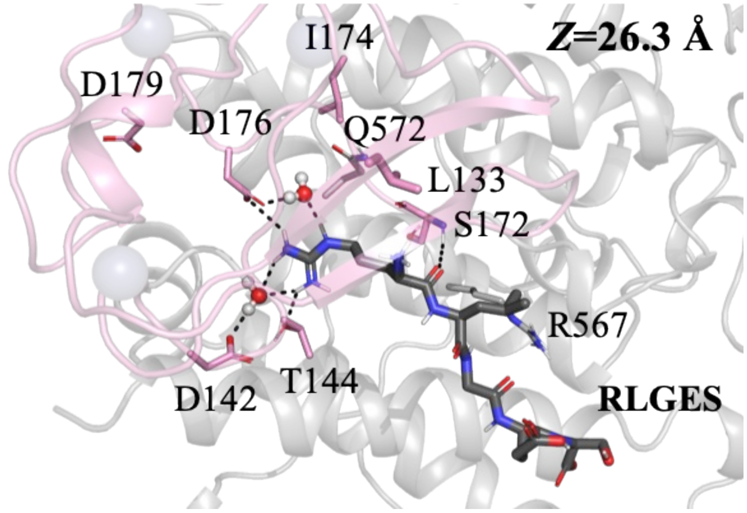

Fig. S5 Representative conformation of initial displacement of RLGES peptide in the binding site. This intermediate conformation corresponds to Z = 26.3 Å along the unbinding pathway. The RLGES peptide is shown as dark grey sticks. The polar interactions between the RLGES peptide, water molecules, and protein residues are represented as black dashed lines.

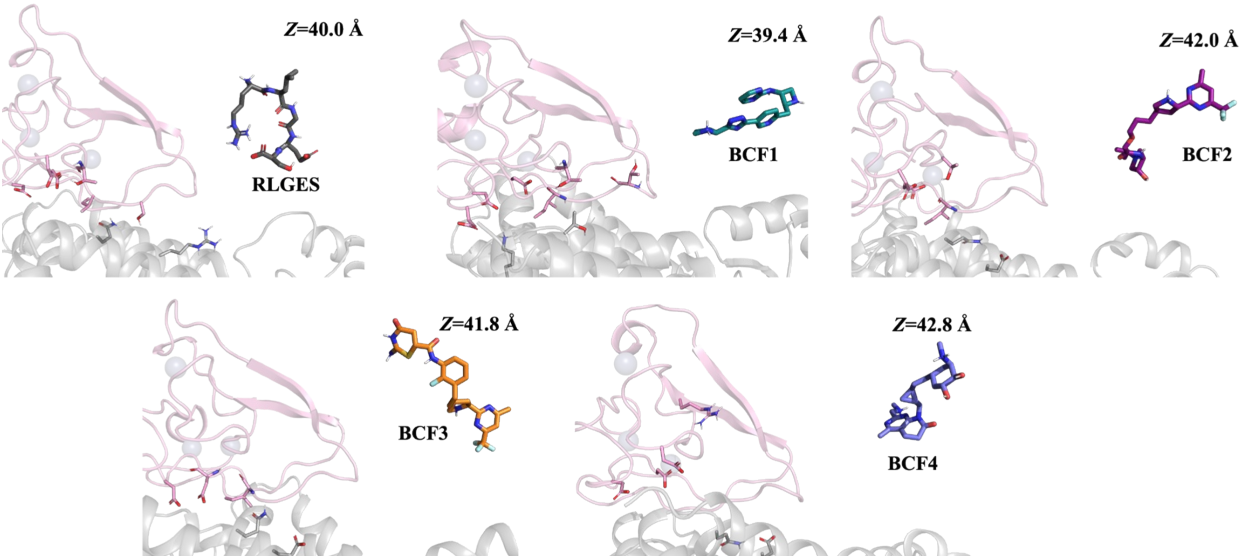

Fig. S6 Representative unbound state conformations of the reference peptide and BCFs. The RLGES peptide, BCF1, BCF2, BCF3, and BCF4, are shown as sticks colored dark grey, teal, purple, orange, and slate sticks, respectively.

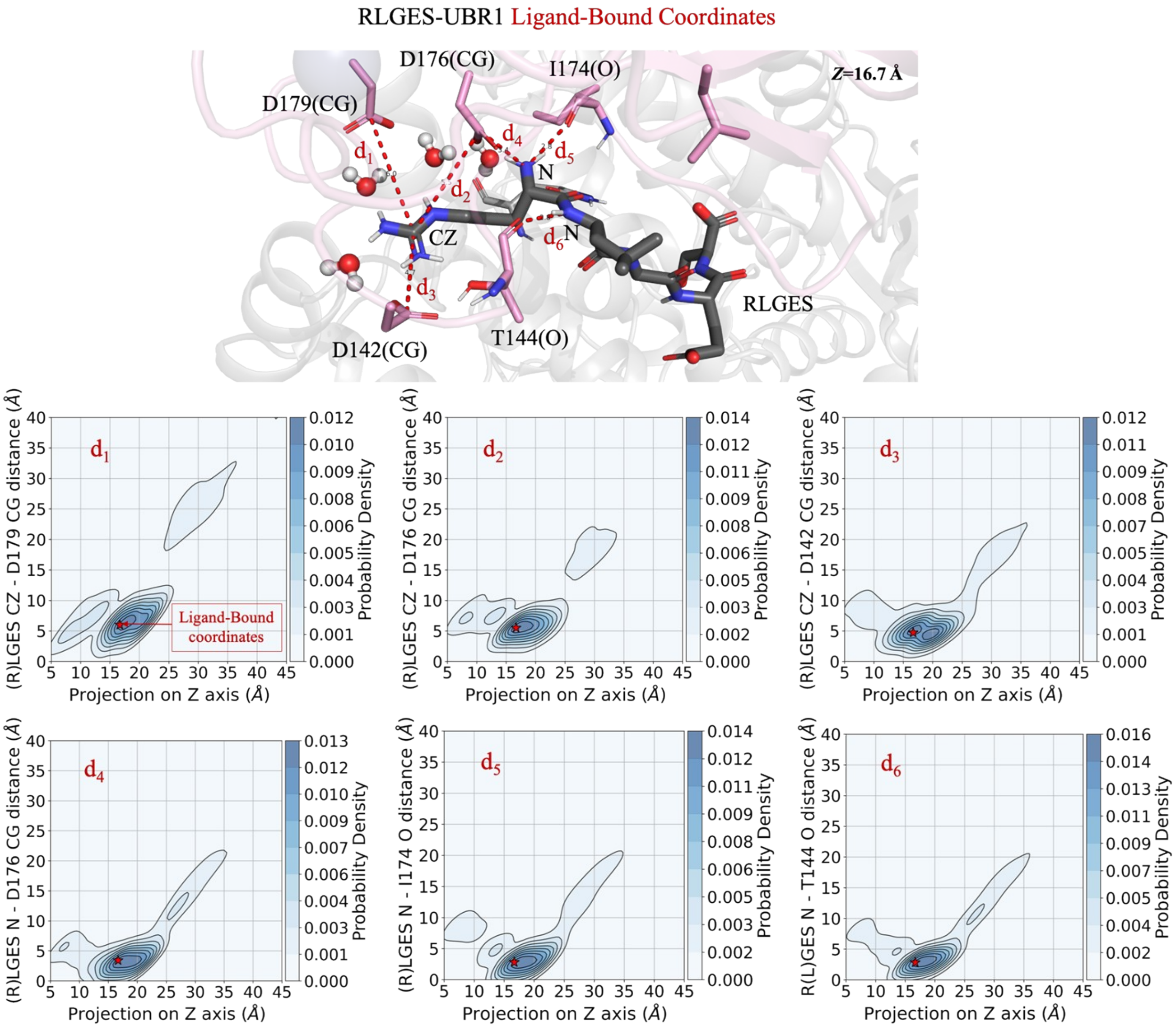

Fig. S7 Population analysis of RLGES-binding site distances during unbinding. The representative bound-state conformation of RLGES is shown together with 2D kernel density plots of distances $d_{1\text{-}6}$ between RLGES and key binding site residues as a function of the projection on the Z axis. The coordinates of the representative bound-state conformation are indicated as a red star. For all distances, the high-probability basin corresponds to the bound region (approximately Z = 14-23 Å), highlighting the stability of the representative bound-state conformation.

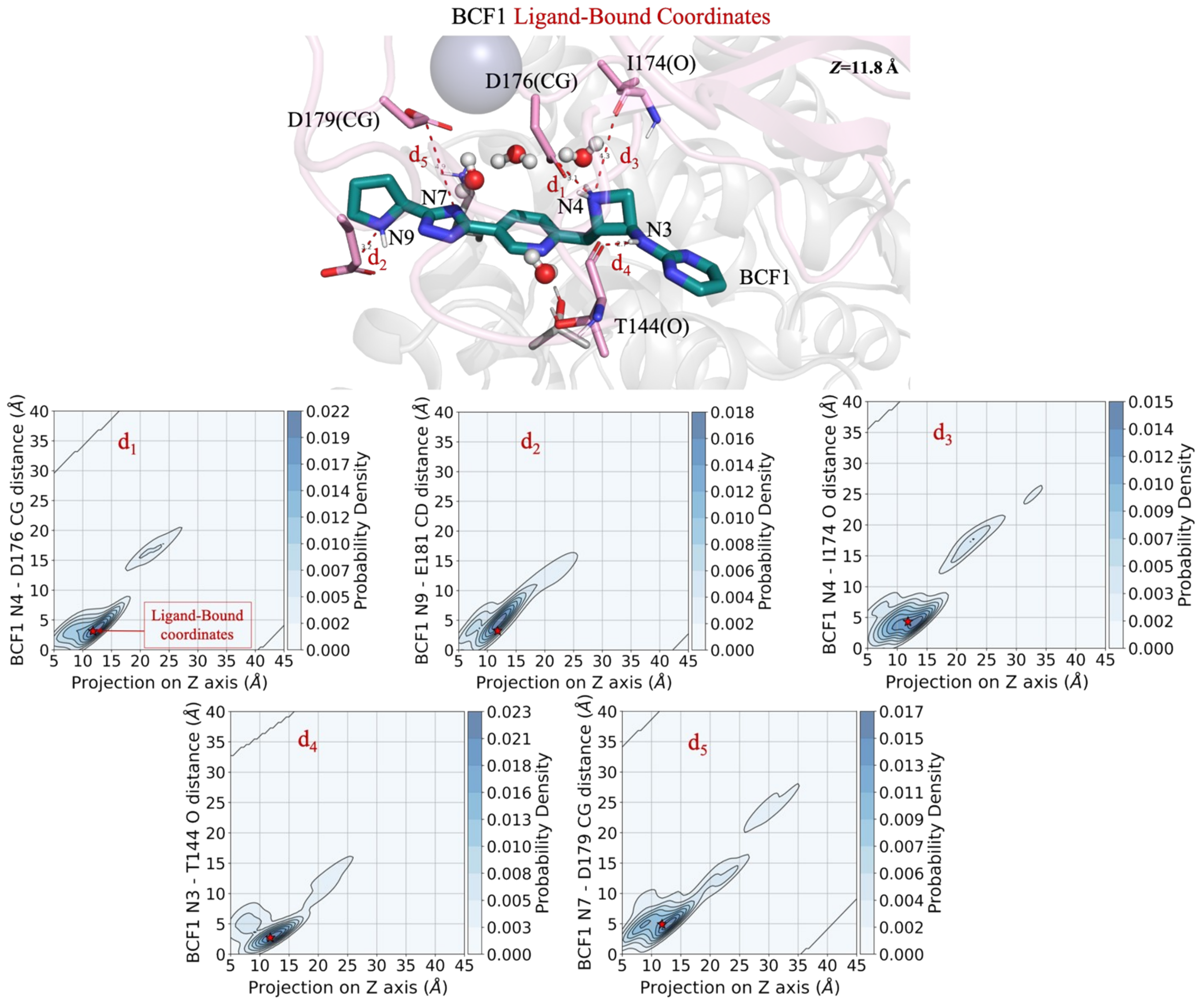

Fig. S8 Population analysis of BCF1-binding site distances during unbinding. The representative bound-state conformation of BCF1 is shown together with 2D kernel density plots of distances $d_{1\text{-}5}$ between BCF1 and key binding site residues as a function of the projection on the Z axis. The coordinates of the representative bound-state conformation are indicated as a red star. For all distances, the high-probability basin corresponds to the bound region (approximately Z = 8-14 Å), highlighting the stability of the representative bound-state conformation.

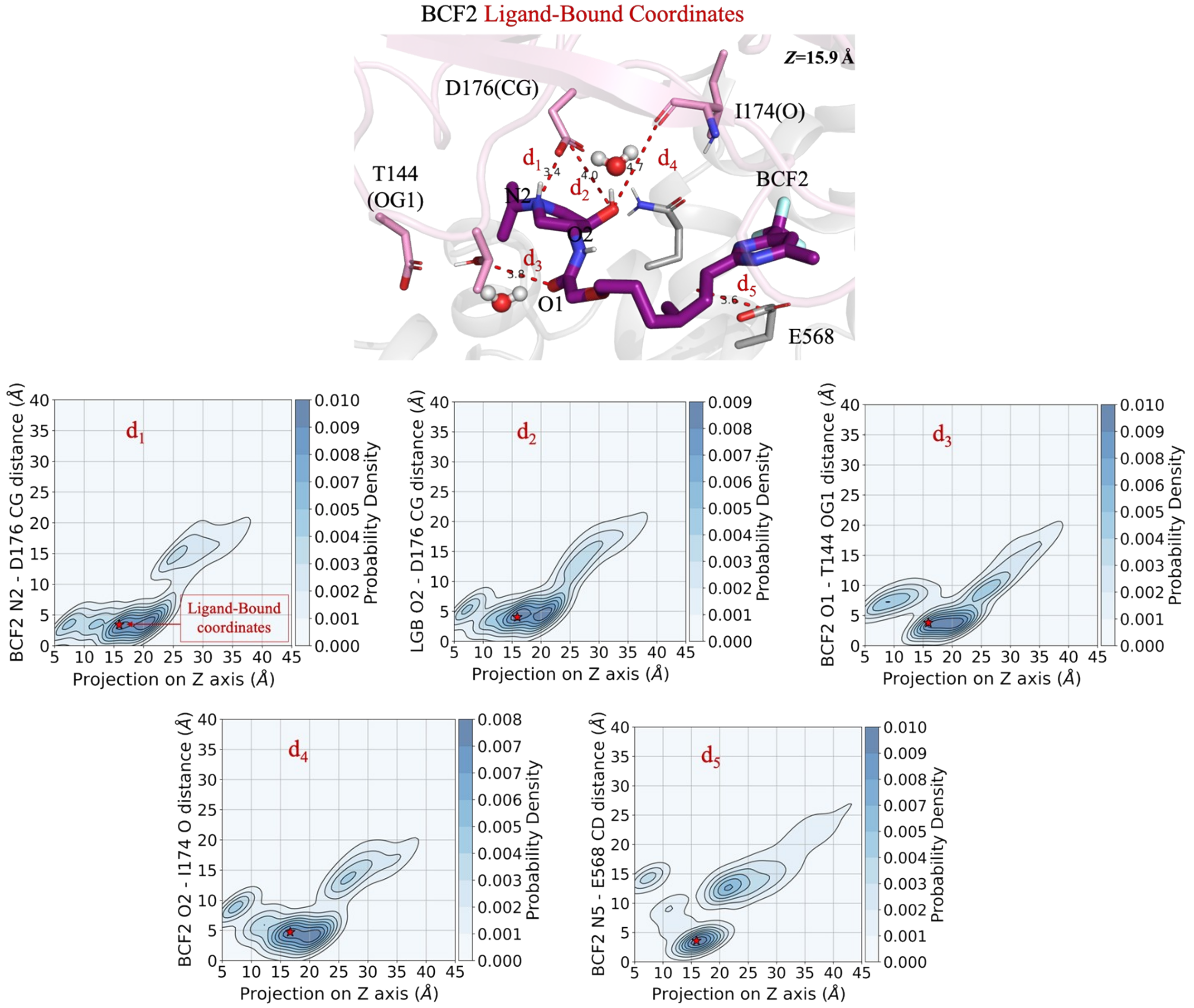

Fig. S9 Population analysis of BCF2-binding site distances during unbinding. The representative bound-state conformation of BCF2 is shown together with 2D kernel density plots of distances $d_{1\text{-}5}$ between BCF2 and key binding site residues as a function of the projection on the Z axis. The coordinates of the representative bound-state conformation are indicated as a red star. For all distances, the high-probability basin corresponds to the bound region (approximately Z = 15-22 Å), highlighting the stability of the representative bound-state conformation.

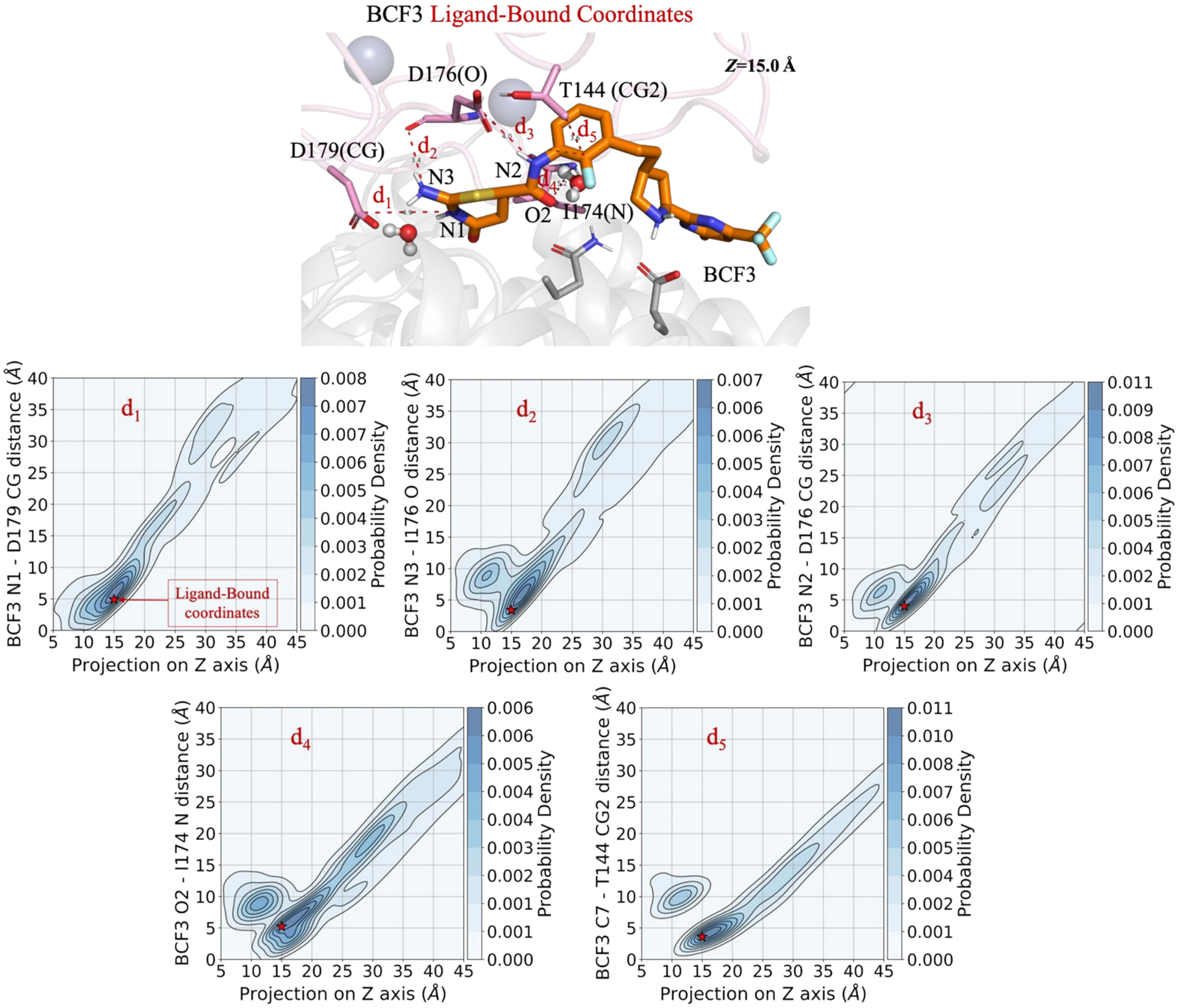

Fig. S10 Population analysis of BCF3-binding site distances during unbinding. The representative bound-state conformation of BCF3 is shown together with 2D kernel density plots of distances $d_{1\text{-}5}$ between BCF3 and key binding site residues as a function of the projection on the Z axis. The coordinates of the representative bound-state conformation are indicated as a red star. For all distances, the high-probability basin corresponds to the bound region (approximately Z = 14-18 Å), highlighting the stability of the representative bound-state conformation.

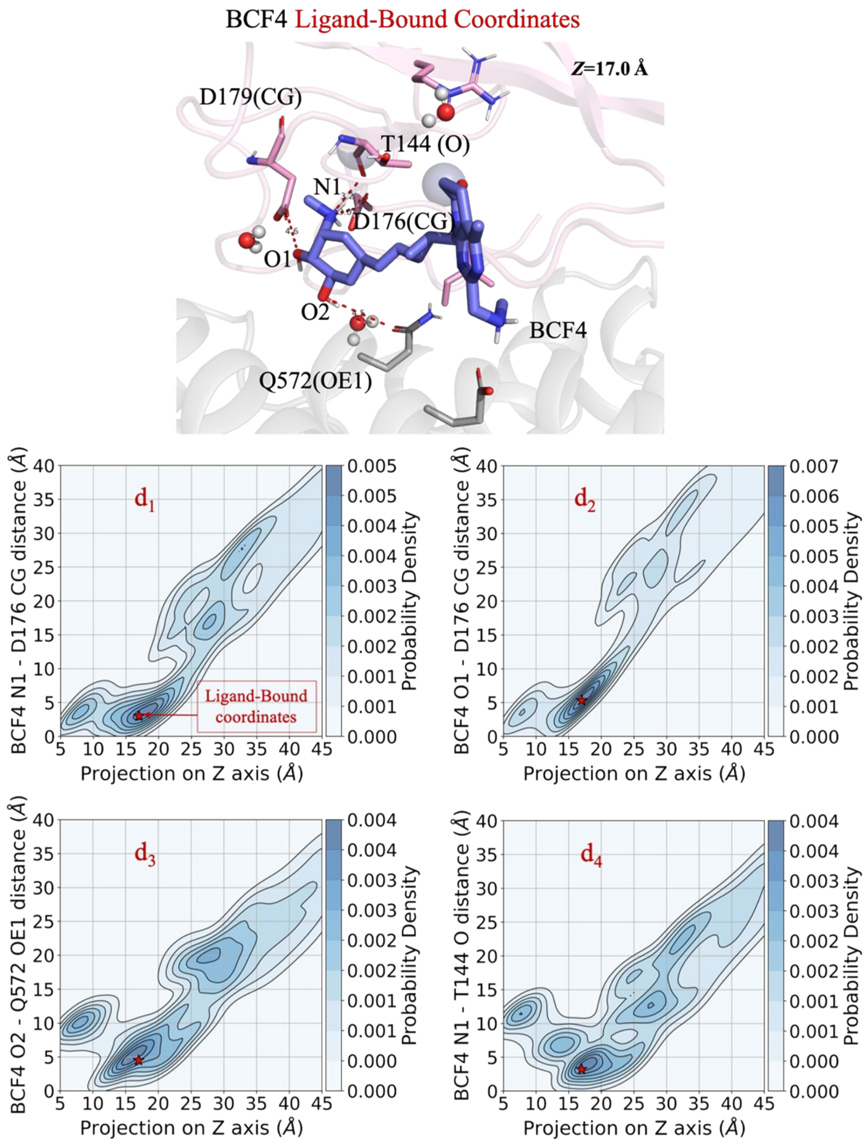

Fig. S11 Population analysis of BCF4-binding site distances during unbinding. The representative bound-state conformation of BCF4 is shown together with 2D kernel density plots of distances $d_{1\text{-}5}$ between BCF4 and key binding site residues as a function of the projection on the Z axis. The coordinates of the representative bound-state conformation are indicated as a red star. For all distances, the high-probability basin corresponds to the bound region (approximately Z = 15-20 Å), highlighting the stability of the representative bound-state conformation.

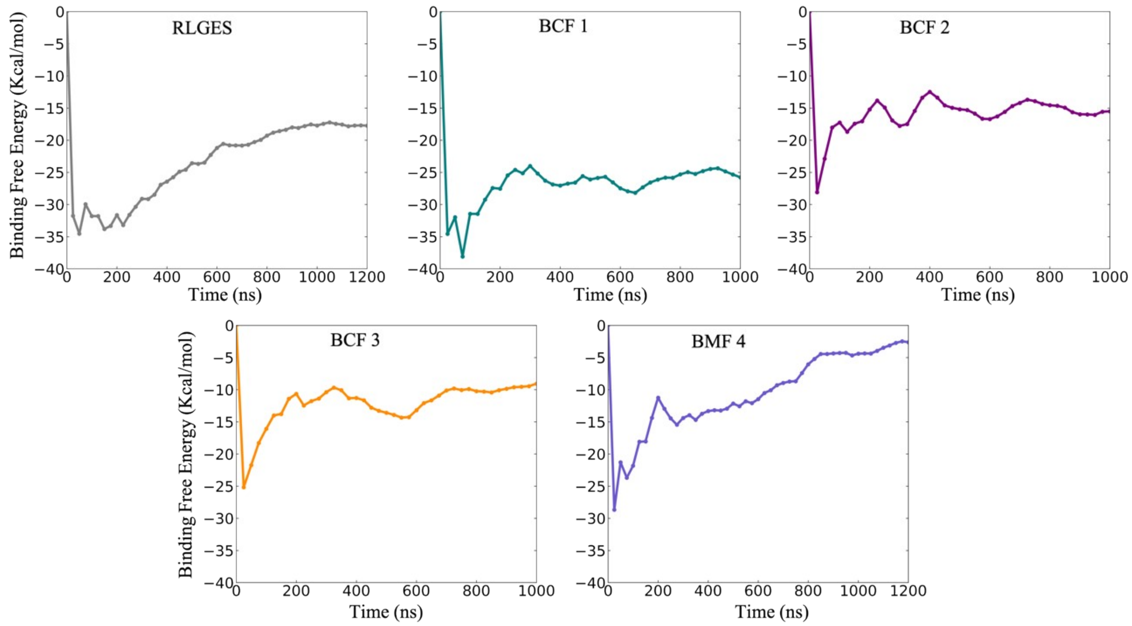

Fig. S12 Convergence of the binding free energy for the N-Degron peptide (RLGES) and the designed BCF 1-4. The lines represent the free energy differences between the bound and unbound state regions along the simulation time. The binding free energies were calculated every 25 ns. In the last part of the simulation (over the last 200 ns), the binding free energies do not change significantly, which indicates convergence. Accordingly, the binding free energy values used in this work were calculated as the average over the last 200 ns of the simulation.

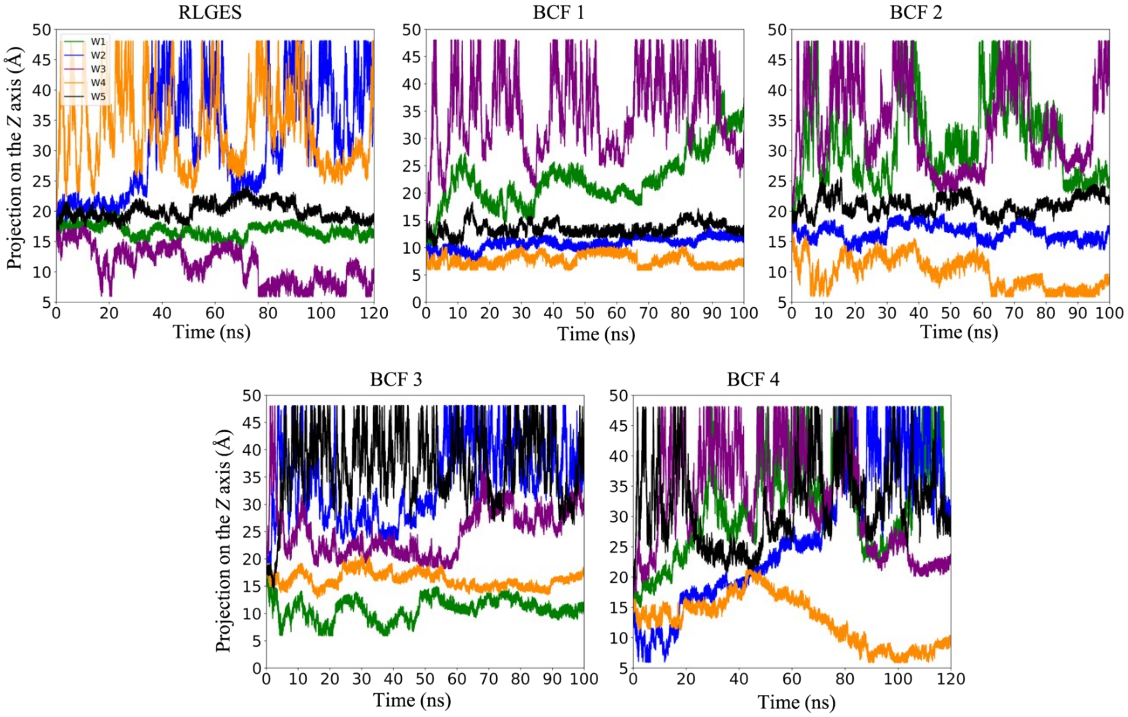

Fig. S13 Evolution of the COM of the BCF 1-4 on the Z axis of the funnel shape over the simulation time. All walker replicas (W1-5) started from the bound state in all systems. The plots show that the bias potential applied during the FM simulations encourages the walkers to sample the binding pathway and explore re-crossing events between bound and unbound states, thereby successfully reconstructing the binding free energy surface.

References

1 Wolber, G.; Langer, T., LigandScout: 3-d pharmacophores derived from protein-bound Ligands and their use as virtual screening filters, *Journal of Chemical Information and Modeling,* 2005, **45** (1), 160-169.

2 *Schrödinger Release 2022-3: Protein Preparation Wizard; Epik, Schrödinger, LLC, New York, NY, 2022; Impact, Schrödinger, LLC, New York, NY; Prime, Schrödinger, LLC, New York, NY, 2022.*

3 Lu, C.; Wu, C.; Ghoreishi, D.; Chen, W.; Wang, L.; Damm, W.; Ross, G. A.; Dahlgren, M. K.; Russell, E.; Von Bargen, C. D.; et al., OPLS4: Improving Force Field Accuracy on Challenging Regimes of Chemical Space, *J Chem Theory Comput,* 2021, **17** (7), 4291-4300.

4 *Schrödinger Release 2022-3: LigPrep, Schrödinger, LLC, New York, NY, 2022.*

5 Shelley, J. C.; Cholleti, A.; Frye, L. L.; Greenwood, J. R.; Timlin, M. R.; Uchimaya, M., Epik: a software program for pK(a) prediction and protonation state generation for drug-like molecules, *J Comput Aided Mol Des,* 2007, **21** (12), 681-691.

6 *Schrödinger Release 2022-3: Glide, Schrödinger, LLC, New York, NY, 2022.*

7 D.A. Case, K. B., I.Y. Ben-Shalom, S.R. Brozell, D.S. Cerutti, T.E. Cheatham, III, V.W.D. Cruzeiro, T.A. Darden, R.E. Duke, G. Giambasu, M.K. Gilson, H. Gohlke, A.W. Goetz,R Harris, S. Izadi, S.A. Izmailov, K. Kasavajhala, A. Kovalenko, R. Krasny, T. Kurtzman, T.S. Lee, S. LeGrand, P. Li, C. Lin, J. Liu, T. Luchko, R. Luo, V. Man, K.M. Merz, Y. Miao, O. Mikhailovskii, G. Monard, H. Nguyen, A. Onufriev, F. Pan, S. Pantano, R. Qi, D.R. Roe, A. Roitberg, C. Sagui, S. Schott-Verdugo, J. Shen, C.L. Simmerling, N.R. Skrynnikov, J. Smith, J. Swails, R.C. Walker, J. Wang, L. Wilson, R.M. Wolf, X. Wu, Y. Xiong, Y. Xue, D.M. York and P.A. Kollman, Amber 2020, *University of California, San Francisco,* 2020.

8 Jakalian, A.; Jack, D. B.; Bayly, C. I., Fast, efficient generation of high-quality atomic charges. AM1-BCC model: II. Parameterization and validation, *J Comput Chem,* 2002, **23** (16), 1623-1641.

9 Limongelli, V., Ligand binding free energy and kinetics calculation in 2020, *Wiley Interdisciplinary Reviews-Computational Molecular Science,* 2020, **10** (4).

10 Raniolo, S.; Limongelli, V., Ligand binding free-energy calculations with funnel metadynamics, *Nature Protocols,* 2020, **15** (9), 2837-2866.

11 Tribello, G. A.; Bonomi, M.; Branduardi, D.; Camilloni, C.; Bussi, G., PLUMED 2: New feathers for an old bird, *Computer Physics Communications,* 2014, **185** (2), 604-613.
